# USP14 is crucial for proteostasis regulation and α-synuclein degradation in human SH-SY5Y dopaminergic cells

**DOI:** 10.1016/j.heliyon.2025.e42031

**Published:** 2025-01-23

**Authors:** Vignesh Srinivasan, Rabah Soliymani, Larisa Ivanova, Ove Eriksson, Nina Peitsaro, Maciej Lalowski, Mati Karelson, Dan Lindholm

**Affiliations:** a Department of Biochemistry and Developmental Biology, Faculty of Medicine, University of Helsinki, P.O. Box 63, FIN-00014, Finland; b Minerva Foundation Institute for Medical Research, Biomedicum Helsinki 2U, Tukholmankatu 8, FIN-00290, Helsinki, Finland; c HiLIFE, Meilahti Clinical Proteomics Core Facility, University of Helsinki, Helsinki, Finland; d Institute of Chemistry, University of Tartu, Ravila 14a, 50411, Tartu, Estonia; e Institute of Molecular Biology and Biochemistry, Department of Gene Expression, Faculty of Biology, Adam Mickiewicz University, Poznań, Poland; f Department of Biomedical and Clinical Sciences, Linköping University, Linköping, Sweden

**Keywords:** USP14, Proteasome, α-Synuclein, pS129 α-Synuclein, Oxidative stress, Autophagy, Phosphorylation

## Abstract

Ubiquitin specific protease-14 (USP14) is critical for controlling proteostasis disturbed in human disorders, including Parkinson's disease (PD). Here we investigated USP14 in the regulation of α-synuclein (α-syn) degradation via the proteasome and autophagy. α-Syn and pS129 α-syn were elevated in *USP14* gene-deleted SH-SY5Y dopaminergic cells with decreased proteasome activity. However, autophagy and coordinated lysosomal expression and regulation pathways were elevated in USP14 lacking cells with higher levels of the transcription factor TFEB. There was an increase in reactive oxidative species (ROS) and elongated mitochondria in USP14 deficient cells and counteracting oxidative stress decreased α-syn levels. Phosphoproteomics revealed that USP14 is phosphorylated at residue S143 that reduces its binding to the proteasome. Re-expression of wild-type and phospho-mimetic S143D-USP14 mutant lowered ROS and α-syn levels in USP14 lacking cells. USP14 is a promising factor to consider in PD to target α-syn through its regulation of proteasomes and oxidative stress in dopaminergic neurons.

## 1 Introduction

Protein quality control is essential for maintaining optimal cell functions and growth. Disturbances in protein homeostasis are associated with several human diseases, including cancer and neurodegenerative disorders. Protein-folding by molecular chaperones, degradation pathways via the ubiquitin-proteasome system (UPS), and autophagy-lysosome pathway (ALP) contribute to optimal proteostasis [[1], [2], [3]]. UPS and ALP can act in conjunction to control the levels of cellular proteins and intra-cellular organelle homeostasis in health and disease [[4], [5], [6]]. However, the precise mechanisms by which these systems are interconnected in the context of cellular physiology, are largely unknown.

Parkinson's disease (PD) is associated with an increase in soluble and aggregation-prone α-syn levels due to defects in protein degradation, and folding mechanisms [7,8]. A hallmark of the disease is the accumulation of α-syn in intracellular aggregates termed Lewy bodies that form within PD-susceptible midbrain dopaminergic neurons. This is accompanied by negative effects on cellular pathways causing disturbed protein handling, mitochondria defects, increased oxidative stress-associated damage, and ER stress [9,10]. The mechanisms by which misfolded α-syn are elevated in PD are not fully understood but defects in proteasome and autophagy machinery are proposed to contribute to the disease.

The association of α-syn with the chaperone Rpn14/PAAF1 was shown to inhibit the 26S proteasome activity in yeast as well as in human cells [11]. α-Syn is further known to undergo various post-transcriptional modifications in cells, including phosphorylation and ubiquitination [12,13]. Among the phosphorylated species of α-syn, the S129 phosphorylated form (pS129 α-syn) has been associated with PD and the aggregation of α-syn [[14], [15], [16], [17], [18]]. Ubiquitination of α-syn is controlled by various E3 ubiquitin ligases that either promote aggregation of α-syn, like SIAH1/2, or enhance its degradation, including CHIP, Parkin and the NEDD4 family of ligases [12]. Furthermore, also the deubiquitinating enzymes (DUBs), USP8, USP13, and UCH-L1 have been shown to reduce the clearance of α-syn [12]. In contrast, little is so far known about the functions of USP14 in PD or in α-syn degradation (see below). Adding to the complexity, the E3 ligases and DUBs are also important in regulation of the ubiquitination status of mitochondria-associate proteins causing alterations in mitophagy that are part of the pathogenesis in PD [19,20].

The DUB, USP14 is an adaptive regulator of the mammalian 26S proteasomes and influences it by multiple mechanisms [[21], [22], [23], [24], [25], [26], [27], [28], [29]]. USP14 associates with the proteasome in a reversible manner and can exert both positive and negative effects on the 26S proteasome activity [23,24]. Ubiquitinated proteins upon binding to USP14/UBP6 (in yeast) accelerate 26S proteasome degradation by inducing sequential structural changes to 19S RP [23,24,30]. Normally in the absence of ubiquitinated substrates, USP14 behaves as an allosteric inhibitor of the proteasome-degradation activity [31]. USP14 upon binding to polyubiquitin chain on substrates, activates the 19S ATPase ring, and induces the alignment of the 20S CP translocation channel [29,30]. This facilitates the entry of unfolded substrates into the 20S translocation channel upon removal of the polyubiquitin chains by USP14 and Rpn11. USP14 exists in a bidirectional relationship with the proteasomes such that the binding of USP14 to the proteasomes, increases its DUB activity 800-fold [21,25]. On the other hand, binding of the UBL-domain of USP14 or other UBL-domain containing proteins such as Rad23 is shown to be enough to activate the 26S proteasome [28,32]. Furthermore, USP14, by removal of ubiquitin chains on protein substrates can antagonize their degradation and stabilize the proteins [26,33].

Recent data show further that USP14 is dynamically involved in the control of various pathways in the cell, such as autophagy and ER stress [34,35]. USP14 has been linked to a range of different diseases such as tumor growth and cancer [[36], [37], [38], [39], [40]], but its role in neurodegenerative disorders has been less studied. A recent study revealed that the USP14 concentration in the cerebrospinal fluid (CSF) of female PD patients was slightly lower than in control patients. Furthermore, a mouse model with inactivation of USP14 showed that autophagy as well as the clearance of α-synuclein were increased particularly in female mice. The effects of USP14 deficiency were associated with S100A8/A9 activation and reduced inflammation in microglia cells. However, the functional roles of USP14 on α-synuclein in neurons in models of PD are so far unclear [41].

In the present work, we have employed human SH-SY5Y dopaminergic cells to investigate the role of USP14 in the regulation of proteostasis pathways and degradation of α-syn. Utilizing gene deletion of *USP14*, we observed that the levels of α-syn, and its S129 phosphorylated form (pS129 α-syn) associated with PD [[14], [15], [16], [17], [18]], were elevated in SH-SY5Y neuronal cells with a concomitant reduction in proteasome activity and increases in ROS. Autophagy and Coordinated Lysosomal Expression and Regulation Pathways (CLEAR) signaling [42] were also increased in the USP14 deficient cells possibly due to compensatory reactions resulting from compromised proteasome. Utilizing phosphoproteomics, we identified that USP14 is phosphorylated at S143 in control SH-SY5Y cells. Molecular modeling studies revealed that the S143 phosphorylation could affect its structure and modulate the N-terminal UBL-domain interactions. In line with this, the phosphomimetic S143D-USP14 mutant showed less interaction with the proteasome as revealed by protein interaction and native-PAGE assays. Re-expression of wildtype USP14 and the S143D-USP14 mutant reduced the ROS in USP14 deficient cells as did the addition of N-acetylcysteine amide (NACA). NACA further decreased α-syn in USP14-deleted cells indicating a role for oxidative stress in α-syn regulation in these cells. These results demonstrate that USP14 may be a potent factor for targeting α-syn and pS129 α-syn levels via influencing proteasomes and oxidative stress in neuronal cells and in PD.

## 2 Materials and methods

### 2.1 Cell culture and transfections

The SH-SY5Y human neuroblastoma cell line has been used as a culture model of dopaminergic neurons in studies of PD [70]. SH-SY5Y cells (CRL-2266, ATCC, USA, RRID:CVCL 0019) were cultured in Dulbecco's Modified Eagle Medium (DMEM) (Gibco, USA) supplemented with 10 % fetal bovine serum (Gibco, USA), 7.5 % NaHCO3, 100 mM NA-glutamine (Gibco, USA) and 100 mM penicillin-streptomycin (Gibco, USA) at 37 °C in 5 % CO_2_.

Cells were transfected with Linear Polyethylenimine 25.000 (PEI) (Polysciences, Inc., USA) or Fugene HD reagent (Promega, USA) following the manufacturer's instructions. For PEI, stock PEI solution was prepared at a concentration of 1 mg/ml. For complexing the DNA with PEI or Fugene, they were combined at a ratio of 1:3 and added onto the cells. Following 24–48 h of transfection, cells were further processed and used in experiments described below.

### 2.2 CRISPR/Cas9 mediated *USP14* gene deletion

*USP14* gene deletion in SH-SY5Y cells was performed utilizing the Clustered regularly interspaced short palindromic repeats (CRISPR) Cas9 endonuclease (CRISPR-Cas9) system as previously described [71]. Briefly, CRISPR design tool (https://crispr.mit.edu) was used to design guide RNAs (gRNAs) targeting the exon 2 of human *USP14* (common exon to most human USP14 transcripts). gRNA USP14_Forward: 5′ CACCGGTGAGCCTTGAATACCATTGG 3′ and gRNA USP14_Reverse: 5′ AAACCCAATGGTATTCAAGGCTCACC 3’ were cloned into pSpCas9(BB)-2A-Puro vector (PX459, 62988, Addgene, USA) using FastDigest BpiI (Thermo Fisher Scientific, USA) and T4 DNA ligase (New England Biolabs, USA). The plasmids were sequenced and SH-SY5Y cells were cultured and transfected as described above. Control cells were transfected with pSpCas9(BB)-2A-Puro vector without USP14 specific gRNAs. At 48 h post-transfection, puromycin-containing growth medium was added, and cells selected for using single-cell clones in 96-well plates to generate stable cell clones. Validation of the USP14 knock-out (KO) cell clones was conducted using immunoblotting with anti-USP14 antibody.

### 2.3 Mutagenesis of USP14

Site-directed mutagenesis was done to generate Serine143 (S143) mutations in human *USP14* gene. at Genome Biology Unit (GBU), University of Helsinki. In brief, the USP14 entry clone from the human ORFeome collaboration library was used to perform mutagenesis and the constructs obtained were transferred into the 2Flag-pDEST-N (118371, Addgene, USA) vector using standard reaction protocol. The S143A-USP14 (loss-of-function) and S143D-USP14 (gain-of function) constructs were further sequenced and employed for cell cultures studies. The 2Flag-pDEST-N vector with no insert was utilized as a negative control for transfections.

### 2.4 Molecular modeling of USP14

*Homology modeling of the structure of the USP14 and generation of USP14 mutant structures.* The 3D structure of the full-length USP14 was constructed using the hierarchical approach to protein structure and function prediction by I-TASSER (Iterative Threading ASSEmbly Refinement) server [72]. The structure with higher C- and TM-scores was selected for further modelling. The structures of the USP14 mutated at S143 residues (S143A and S143D) were generated using the Maestro 13.0 interface of the Schrödinger Software. The structure of the USP14 phosphorylated at S143 residue was generated using PyTMs plugin in The PyMOL Molecular Graphics System, Version 3.0 Schrödinger, LLC [73].

The 3D structure of the USP14 obtained by homology modelling was treated before further molecular dynamics (MD) simulation using Schrodinger Protein Preparation Wizard [74]. Thereafter, the prepared structure of the protein was minimized using MD simulation by Desmond program package of Schrödinger LLC [75]. The structure optimized by MD simulation was used for further modelling.

*Molecular dynamics (MD) simulations*. The MD simulations were carried out using the Desmond program package of Schrödinger LLC [75,76]. The MD simulations were performed similarly as described previously [77]. The analysis of the MD trajectories was performed using the Simulation Interaction Diagram tool implemented in Desmond molecular dynamics package [76].

### 2.5 Immunoblotting

Cells were washed with ice-cold PBS, twice and lysed in Radioimmunoprecipitation assay buffer (RIPA) buffer containing 150 mM NaCl, 1 % Triton-X-100, 0.5 % sodium deoxycholate, 1 % SDS, 50 mM Tris-HCl, pH 7.4 supplemented with protease inhibitors (Roche, Switzerland) and phosphatase inhibitor (Phosphostop, Roche, Switzerland) [35,40]. Lysates were sonicated, centrifuged, and the supernatant protein concentration measured using BCA protein assay kit (Pierce, Thermo Fisher Scientific, USA). Equal amounts of proteins were separated on denaturing SDS-PAGE and blotted onto nitrocellulose membrane filters (Sartorius). The membranes were blocked and incubated with the primary antibodies overnight at 4 °C with gentle agitation. The primary antibodies used included: USP14 (J6111-6D6, Sigma, Germany), α-synuclein (2642, CST, USA), pS129-α-synuclein (EP1536Y, Abcam, GB), 20S CP subunits cocktail (BML-PW8195, Enzo Lifesciences, USA), K48-linkage specific polyubiquitin (4289, CST), PSMD2 (sc-271775, SantaCruz, USA), PSMC2 (14395, CST), UCHL5 (sc-271002, SantaCruz, USA), TFEB (37785, CST, USA), GBA1 (sc-166407, SantaCruz, USA), GABARAP (13733, CST, USA), LC3B (3868, CST, USA), p62/SQSTM1 (P0067, Sigma, Germany), beta-Actin (A2066, Sigma, Germany), GAPDH (MAB374, Millipore, Germany), OXPHOS cocktail (MS604, Mitosciences, USA – Currently Abcam, GB).

Following incubation overnight, horse-radish peroxidase conjugated secondary antibody (1:2500, Jackson Immunoresearch Laboratories, USA) were added and the membranes incubated for 1 h at room temperature with gentle agitation. Protein signals were detected using enhanced chemiluminescence substrate (Pierce, Thermo Fisher Scientific, USA). Immunoblots were quantified with ImageJ (NIH, Bethesda, USA) quantification software.

For immunoblotting of pS129-α-synuclein, the samples were heated and separated on BioRad 4–20 % gradient gels and transferred onto nitro-cellulose membranes. Upon transfer, the membranes were fixed immediately in 0.4 % PFA diluted in TBS. The membrane was then washed with TBS-T, blocked with 5 % milk diluted in TBS-T and incubated in pS129-α-synuclein antibody for 1 h, RT with gentle agitation. Following this, membranes were washed with TBS-T and incubated with HRP-conjugated anti-rabbit secondary antibodies for 1 h, RT with gentle agitation. Post antibody incubation, membranes were washed with TBS-T and imaged using enhanced chemilumisescence substrate as above.

### 2.6 Immunoprecipitation

Cells were cultured and lysed in IP-lysis buffer containing 50 mM Tris-HCl pH 7.7, 150 mM NaCl, 1 % NP-40, and 0.5 % sodium deoxycholate for 15min on ice followed by centrifugation at 10,000×*g* for 10min at 4 °C [35]. The supernatant was collected, and protein concentration was measured using BCA assay (Pierce, Thermo Fisher Scientific, USA).

The antibody-bead complexes were centrifuged, pelleted, and washed with IP-lysis buffer thrice. Immunoprecipitated proteins were eluted by the addition of 25 μl 2× denaturing Laemmli buffer and heated to 98 °C for 5 min. The eluates were then subjected to conventional denaturing SDS-PAGE and analyzed using immunoblotting for the indicated antibodies. The total cell lysates served as the input controls.

### 2.7 Native-PAGE to assess in-gel proteasome activity assay and protein complex composition

20S CP and 26S/30S proteasome chymotrypsin-like activity, and proteasome complexes were analyzed using in-gel proteasome activity assay and immunoblotting followed by Native-PAGE, as described before [40,44,78,79]. Control and USP14-deleted SH-SY5Y cells received different treatments and lysed in Over Kleeft (OK) lysis buffer (50 mM Tris-HCl pH7.5, 2 mM DTT, 5 mM MgCl_2_, 10 % glycerol, 2 mM ATP, and 0.05 % Digitonin) by incubation on ice for 20min with intermittent vortexing. Lysates were then centrifuged at 27,670×*g* for 20min at 4 °C, protein concentration were estimated as above, and an equal amount of protein was separated on Tris-Borate PAGE in a running buffer comprised of Tris-borate buffer supplemented with EDTA-Na2, ATP and MgCl_2_. Gels were run at a constant voltage of 120 V approximately 2 h 30min in the cold room. Following this, the gels were either transferred to a buffer containing in-gel activity substrate or buffer for performing immunoblotting.

For in-gel activity assay, gels were incubated for 15min, 37 °C in the substrate buffer (50 mM Tris-HCl pH 7.4, 5 mM MgCl_2_, 1 mM ATP) supplemented with 100 mM of Suc-Leu-Leu-Val-Tyr-AMC (Bachem, Switzerland) substrate for chymotrypsin-like activity of 20S CP. Gel imaging was performed by exposing them to an excitation wavelength of 380 nm and emission wavelength of 460 nm. Upon imaging, the gels were incubated again for 15 min 37 °C in the above-mentioned substrate-containing buffer added with SDS to open the gating of free 20S CP [44]. Furthermore, the gels were imaged again to obtain activated 20S CP activity in the presence of SDS.

For immunoblotting gels were incubated in conventional Tris-Glycine running buffer containing SDS for 10 min with gentle shaking and then transferred to the Tris-Glycine transfer buffer containing methanol. Standard protocol for wet-transfer was performed for 16 h, 20 V at 4 °C followed by incubation with 20S (BML-PW8195, Enzo Lifesciences, USA), anti-USP14 antibody (J6111-6D6, Sigma, Germany).

### 2.8 *In-vivo* DUB catalytic activity assessment using the Ub-VME substrate

The labelling assay for DUB activity was performed as described earlier [22,35]. SH-SY5Y cells were transfected with Flag-tagged WT-USP14 or S143A-USP14 or S143D-USP14. Cells were lysed after 24 h using 50 mM Tris (pH 7.4), 250 mM sucrose, 5 mM MgCl2, 1 mM DTT and 1 mM ATP for 1 h at 4 °C, and 30 μg of cell lysates were incubated with 2 mM ubiquitin vinyl methyl ester (Enzo Life Science) for 1 h at 37 °C. The reaction was stopped by addition of Laemmli buffer and samples were analyzed by SDS-PAGE immunoblotting as above with Flag antibody to visualize the UbVME-bound Flag-tagged USP14.

### 2.9 Sample preparation, protein digestion, and nano LC-MS/MS for global and phospho-proteomics

For phospho-proteomic studies control SH-SY5Y cells were transfected with Flag-WT-USP14 and cultured for 48 h and lysed in IP-lysis buffer. Flag-WT-USP14 was immunoprecipitated utilizing Anti-Flag M2 (F1804, Sigma, Germany) antibody and eluted with 200 mM Glycine pH 2.0. Glycine-eluted samples were neutralized with 1.5 M Tris-HCl pH 8.4 and further processed for trypsin digestion and LC-MS analysis as below.

For global proteomic analysis, control and USP14-deleted cells were cultured for 48 h, trypsinized and collected by centrifugation, followed by homogenization using lysis buffer containing 8 M urea, 100 mM ammonium bicarbonate, 2 % sodium deoxycholate, 0.1 % Octyl α- D -glucopyranoside, and five cycles of vortexing and bath sonication. Proteins were reduced and alkylated using tris (2-carboxyethyl) phosphine (TCEP) and iodoacetamide to a final concentration of 5 mM and 50 mM respectively and incubation in the dark for 30min.

10 μg of protein were washed 8 to 10 times with 8M urea, 100 mM ammonium bicarbonate in Amicon Ultra-0.5 centrifugal filters using a modified FASP method [80]. Lysine-C endopeptidase solution (121-05063, FujiFilm Wako, Japan) in a ratio of 1:50 w/w was added to the protein lysates in about 4 M urea/100 mM ammonium bicarbonate and incubated overnight at room temperature (RT). The peptide digests were collected by centrifugation and trypsin solution was added in a ratio of 1:50 w/w in 50 mM ammonium bicarbonate and incubated overnight at RT. The peptide samples were cleaned using Pierce™ C18 reverse-phase tips (Thermo Scientific, USA). Dried peptide digest was re-suspended in 0.3 % TFA and sonicated in a water bath for 3min before injection. Digests were analyzed using nano-LC-MS/MS. The peptides were separated by Ultimate 3000 LC system (Dionex, Thermo Scientific, USA) equipped with a reverse-phase trapping column RP-2TM C18 trap column (0.075 × 10 mm, Phenomenex, USA), followed by analytical separation on a bioZen C18 nano column (0.075 × 250 mm, 2.6 μm particle size; Phenomenex, USA). The injected sample analytes were trapped at a flow rate of 5 μl x min^−1^ in 100 % of solution A (0.1 % formic acid). After trapping, the peptides were separated with a linear gradient of 125min comprising 110min from 3 % to 35 % of solution B (0.1 % formic acid/80 % acetonitrile), 6min to 45 % B, and 4min to 95 % of solution B. Each sample run was followed by an empty run to reduce the potential sample carryover from previous runs.

LC-MS acquisition was done using the mass spectrometer (Thermo Q Exactive HF) settings as follows: The resolution was set to 120,000 for MS scans, and 15000 for the MS/MS scans. Full MS was acquired from 350 to 1400 *m/z*, and the 15 most abundant precursor ions were selected for fragmentation with 45 s dynamic exclusion time. Ions with 2+, 3+, 4+, and 5+ charges were selected for MS/MS analysis. Maximum IT were set as 50 and 25 ms and AGC targets were set to 3 e^6^ and 1 e^5^ counts for MS and MS/MS respectively. Secondary ions were isolated with a window of 1 *m/z* unit. Dynamic exclusion was set with a duration of 45sec. The NCE collision energy stepped was set to 28 kJ mol–1.

### 2.10 Proteomic data and bioinformatic analysis

Following LC-MS/MS acquisition, raw files were qualitatively analyzed by Proteome Discoverer (PD), version 2.5 (Thermo Scientific, USA). The identification of proteins by PD was performed against the human protein database (release 2023_1 with 20434 entries) using the built-in SEQUEST HT engine. The following parameters were used: 10 ppm and 0.02 Da were mass error tolerance values set for MS and MS/MS, respectively. Trypsin was used as the digesting enzyme, and two missed cleavages were allowed. The carbamidomethylation of cysteine residues was set as a fixed modification, while the oxidation of methionine, deamidation of asparagine and glutamine, and phosphorylation of Serine and threonine were set as variable modifications. The false discovery rate was set to less than 0.01 and a peptide minimum length of six amino acids. Label-free quantification was done using unique peptides in Precursor Ion Quantifier. Differentially expressed proteins (DEPs) were identified based on the number of unique peptides used for label-free quantitation (≥2), at the FDR <0.01 and the fold change (FC) from averaged, normalized protein intensities |≥1.5|, utilizing p ≤ 0.05 by ANOVA in all comparisons, serving as inputs for Ingenuity Pathway Analysis (Qiagen™). The proteomics data were deposited to ProteomeXchange Consortium repository via MassIVE platform under the PXD047270 identifier.

### 2.11 MitoTracker staining and Image analysis

Cells were cultured on μ-Slide 8-well ibiTreat plates (80806, Ibidi, Germany) and stained with 250 nM MitoTracker DeepRed FM (Thermo Fisher Scientific, USA) for 30min at 37 °C. Following the staining, the culture medium was replaced with FluoroBrite™ DMEM (Gibco, USA) imaging medium. The stained live-cells were imaged utilizing Nikon Eclipse Ti-E inverted widefield microscope with full environmental chamber, Hamamatsu Orca Flash 4.0 V2 B&W camera for fluorescence and Lumencor Spectra X light engine (Biomedicum Imaging Unit, Medicum, University of Helsinki).

Mitochondria branch length analysis was performed utilizing the ImageJ (NIH, Bethesda, USA) macro – Mitochondria Network Analysis (MiNA) [81]. In each biological experiment, around 60–100 cells from different regions of the culture wells were analyzed for each sample and analyzed for differences in mean branch length parameter provided by MiNA. Briefly, pre-processing protocols provided by the macro were applied, and mitochondrial branches were skeletonized for analysis by MiNA. The changes in mitochondrial branch length measured as mean branch length parameter in the MiNA macro was utilized as a measure of change in mitochondria fusion/fission between control and USP14-deleted cells.

### 2.12 CellROX green staining

Cells were cultured and stained with 5 μM CellROX green reagent (Thermo Fisher Scientific, USA) for 30min at 37 °C. Following the staining, the culture medium was replaced with FluoroBrite™ DMEM (Gibco, USA) imaging medium and imaged with EVOS FL cell imaging system (Thermo Fisher Scientific, USA).

For quantification of intensity, region of interest selection was made around each cell and intensity measured by the measure tool in ImageJ. Background intensity was measured and subtracted from the cell intensity to acquire a more accurate representation of CellROX staining intensity in the cell.

### 2.13 Measurement of mitochondrial respiratory capacity

To study mitochondrial oxygen consumption rate (OCR), Control and USP14 deleted cells were plated on a Seahorse 96-well plate and cultured for 48 h. As described before, basal Oxygen Consumption Rate (OCR) utilizing the Seahorse XFe96 analyzer (Seahorse Bioscience, Boston, MA, USA) [82] and the measurements were plotted as histogram.

### 2.14 Transmission electron microscopy (TEM)

For thin-section TEM, Control and USP14 deleted cells were cultured for 48 h on coverslips and fixed using 100 mM NaPO4, pH 7.4, 2 % Glutaraldehyde (EM-grade, Sigma Aldrich, Germany) for 24hr, 4 °C. Post-fixation, the cells were Osmicated utilizing Osmium tetroxide, dehydrated in ethanol series and acetone followed by gradual infiltration with Epon (TAAB 812) [83,84]. 60 nm sections cut parallel to the coverslips were post-stained with uranyl acetate and lead citrate. Samples were then imaged utilizing the transmission electron microscope Jeol JEM-1400 electron microscope equipped with an Orius SC 1000B bottom-mounted charge-coupled device camera (Gatan, USA) at acceleration voltage of 80 kV.

### 2.15 Statistical analysis

Depending on the experimental design, statistical analysis was performed utilizing Student's t-test or one-way ANOVA. p-value, p < 0.05 was considered as statistically significant. Statistical analysis and the histogram plots were generated using GraphPad PRISM software.

## 3 Results

To investigate the role of USP14 in neuronal cell processes related to PD, we employed CRISPR/Cas9 to target the *USP14* gene in human SH-SY5Y dopaminergic cells. Guide RNAs (gRNAs) were used against the common exon 2 in *USP14* whereas control cells undergoing the same procedure received control Cas9 vector with no guide RNA (Fig. S1A). Single-cell clones obtained were subsequently grown and analyzed by immunoblotting using a specific anti-USP14 antibody, as described in the methods. Cell clones with gene deletion of *USP14* showed no expression of the corresponding protein (Fig. S1A), and the cells were investigated further along with similarly treated control cells.

### 3.1 The 26S proteasome and 20S CP chymotrypsin-like activity are reduced in cells lacking USP14

**Fig. 1:**
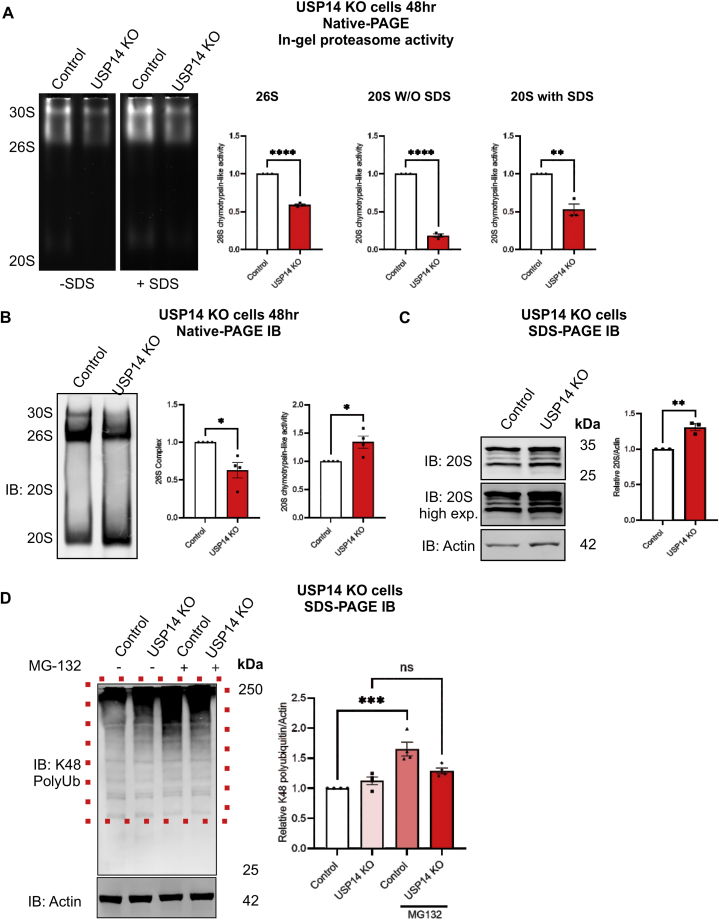
The 26S Proteasome activity and related protein complexes are reduced in USP14-deleted cells. Control SH-SY5Y and USP14-deleted cells were cultured for 48 h as described in Methods (A, B, C). They were then treated for 5 h with 20 μM MG132, or DMSO as negative control, and analyzed further as indicated below. (A) Native-gel electrophoresis followed by in-gel activity assay was done as described in Methods for 26S/30S proteasome and 20S CP chymotrypsin-like activity. Left panels, UV-exposed native gel in the presence or absence of SDS to detect the activity of free 20S CP. Right panels, quantification of the densitometry ratio of 26S/30S proteasome, 20S CP activity without (W/O) or with SDS in USP14-deleted cells normalized to controls. Values are means ± S.E.M. ∗∗p ≤ 0.01, ∗∗∗p ≤ 0.001, ∗∗∗∗p ≤ 0.0001, n = 3. (B) Native-gel electrophoresis followed by immunoblotting using a 20S antibody cocktail. Right panels, quantification of the densitometry ratio of 26S/30S and 20S complex in USP14-deleted cells normalized to controls. Values are means ± S.E.M. ∗p ≤ 0.05. n = 4. (C) Immunoblotting using the antibody for 20S CP subunit proteins. Right panel, quantification of five 20S CP protein subunits normalized to β-actin. (D) Immunoblotting for K48-linked polyubiquitin chains antibody. Red-colored dotted box indicates the K48-polyubiquitin smear region. Right panel, quantification of the K48-polyubiquitin smear normalized to β-actin. (C–D) Values are means ± S.E.M. ∗∗p ≤ 0.01, ∗∗∗p ≤ 0.001, n = 3–4. (A–C) p-value was calculated by Student's t-test and (D) one-way ANOVA.

Previous studies have shown that ubiquitinated proteins upon binding to USP14/UBP6 (in yeast) accelerate proteasome-dependent degradation via activation of the 19S RP ATPase ring and alignment of 20S CP translocation channel [23,24,30]. The 19S RP and 20S CP subcomplexes assemble to form the mature single or double-capped 26S/30S proteasomes that performs ATP- and ubiquitin-dependent protein degradation. 20S CP can act as a standalone complex possessing chymotrypsin-like peptidase activity towards proteins with intrinsically disorders regions (IDR) [43], in a ubiquitin-independent manner. We studied the status of proteasomes in control and USP14 lacking cells cultured in-vitro. Cell lysates were collected from cells in culture for either 24 h (Fig. S1B) or 48 h (Fig. 1A) and subjected to in-gel activity assay using succinyl-LLVY-AMC fluorogenic substrate to assess chymotrypsin-like peptidase activity of 26S/30S proteasome holoenzymes and standalone 20S CP.

Data revealed a downregulation in 26S/30S proteasome activity in USP14-deleted cells compared with controls that were most prominent at 48 h (Figure S1B and Fig. 1A). Examining the free 20S CP chymotrypsin-like peptidase activity in the presence or absence of 0.02 % sodium-dodecyl sulfate (SDS) to relieve the 20S CP gating [44] revealed a reduction in basal 20S CP activity in USP14 lacking cells, which was abrogated following treatment with SDS (Figure S1B and Fig. 1A). 0.02 % SDS relieves the gating of 20S CP by N-termini of α-subunits, which mimics the relief attained by binding to the AAA-ATPase ring of the 19S RP [44]. We then examined the 26S and free 20S CP complex amount utilizing native PAGE immunoblotting (IB) for a 20S antibody cocktail. Results showed that the amount of 26S proteasome complex was downregulated, whilst the free 20S CP complex was increased in USP14 lacking cells prominently at 48 h (Fig. 1B and Fig. S1C).

We next employed the 20S antibody cocktail for immunoblotting with SDS-PAGE to corroborate changes with individual subunits and noted an increase in the 20S CP subunits α5/α7, β1, β5, and β7 in USP14 lacking cells compared with controls (Fig. 1C). Notably, there were no changes in levels of the 19S RP subunits, PSMD2 (Rpn1 in yeast) or in PSMC2 (Rpt1 in yeast) that are known to bind USP14 [21] (Fig. S1D). UCHL5 is another proteasome-associated DUB, but it was not altered in the USP14-deleted cells (Fig. S1E).

Protein substrates targeted for degradation by the 26S proteasome carry lysine-48 (K48) linked ubiquitin chains [45] that can be visualized on immunoblots. Inhibition of the proteasomes using MG132 in control cells showed a significant accumulation of K48-linked polyubiquitinated proteins shown as a smear compared to control cells treated with DMSO (Fig. 1D, red-dotted box). This increase in K48-linked polyubiquitinated smear in response to MG132 was not significant in USP14-deleted cells treated with MG132 compared to USP14-deleted cells treated with DMSO (Fig. 1D, red-dotted box). Taken together the results show that USP14 lacking cells have a reduced chymotrypsin-like peptidase activity of the 26S proteasomes and 20S CP, and a lower amount of ubiquitin-dependent proteasomal degradation.

### 3.2 Autophagy flux is increased in USP14-deleted cells

**Fig. 2:**
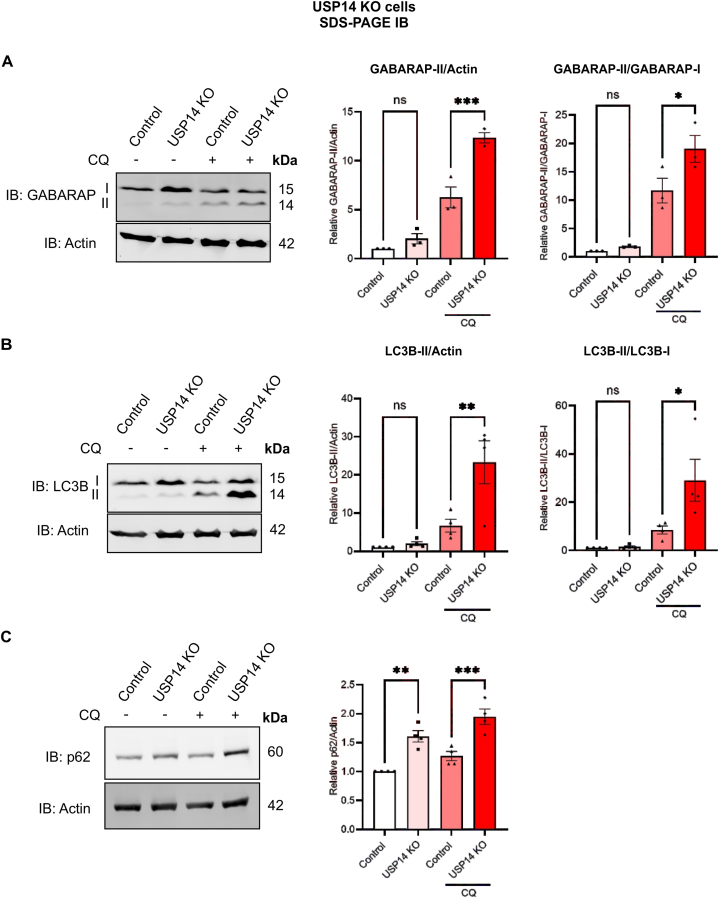
Increase in autophagy flux in USP14-deleted cells. Control and USP14-deleted cells were treated with 200 μM chloroquine diphosphate (CQ) for 4 h to inhibit autophagic flux and analyzed by immunoblotting. (A) Left panel, immunoblot of GABARAP. Right panels, quantification of the densitometry ratio of GABARAP-II normalized to β-actin or GABARAP-I. (B) Left panel, immunoblot of LC3B. Right panels, quantification of the densitometry ratio of LC3B-II normalized to β-actin or LC3B-I. (C) Left panel, immunoblot of p62. Right panel, quantification of the densitometry ratio of p62 normalized to β-actin. Values are means ± S.E.M. ∗∗p ≤ 0.01, ∗∗∗- p ≤ 0.001, n = 3–4. (A–C) p-value was calculated by one-way ANOVA.

The proteasome and autophagy network exist in a tight crosstalk with compensatory responses following defects in either of the systems [46], and some studies indicate that USP14 has a role in this process [[47], [48], [49]]. We therefore studied how autophagy is affected in these USP14-deficient cells by analyzing the ATG8 proteins, LC3Bs, and GABARAPs that are lipidated and recruited to autophagosomes during the process of autophagy. To assess the autophagy flux, cells were treated with chloroquine diphosphate (CQ), which inhibits the fusion of lysosomes with autophagosomes and results in the accumulation of the lipidated ATG8s. Data showed that the lipidation of GABARAP (GABARAP-II) (Fig. 2A) and LC3B (LC3B-II) (Fig. 2B) was increased in cells lacking USP14 which became more prominent in the presence of CQ. Together this indicates that the autophagy flux is enhanced in cells devoid of USP14 compared with controls.

The autophagy receptor SQSTM1/p62 is often used to study the status of autophagy along with changes in ATG8 proteins [50]. We observed that the p62 levels are increased in USP14-deleted cells compared with controls (Fig. 2C) and inhibiting the autophagy flux using CQ lead to a further elevation of p62 in cells. These results suggest a complex regulation of p62 in the USP14-deleted cells likely by both autophagy and proteasomes.

### 3.3 USP14-deleted cells show increases in CLEAR signaling and TFEB

**Fig. 3:**
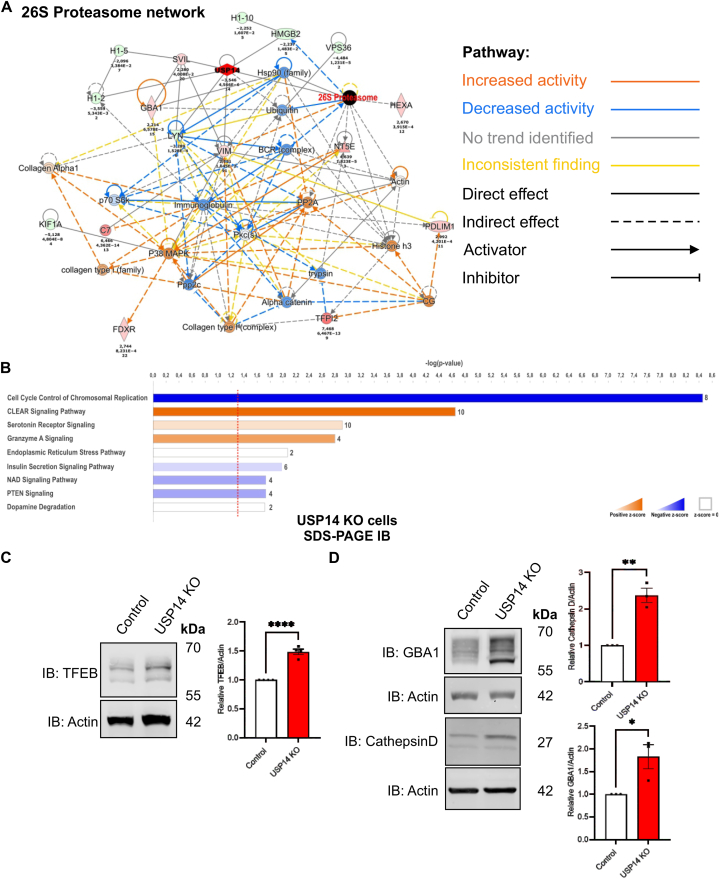
Cellular proteins and signaling networks are altered in USP14-deleted cells. Proteomic analyses of control and USP14-lacking cells were done as described in Methods. (A) Network of interacting proteins related to the proteasome. The USP14 associated node is highlighted by red diamond and the 26S proteasome node by black circle. The color-coding describing changes in activity (red, blue) and direct of interaction is given on the right. The values below distinct proteins refer to changes in expression between USP14 deleted and control cells. (B) Top canonical pathways identified have been plotted as histograms with the number of proteins in each pathway shown to the right. The data was generated using the IPA software from the list of differentially expressed proteins (DEPs) between USP14ablated and control cells using n = 4 biological repeats. The entire list of DEPs is provided in Table S1. The plot is represented as a fold change in p-value on the Y-axis and a threshold of p-value ≥1.3 was considered significant. DEP- Differentially expressed protein, IPA- Ingenuity pathway analysis. (C–D) Cell lysates from control and USP14-deleted cells were subjected to immunoblotting as shown below. (C) Upper panel, immunoblot TFEB. Lower panel, quantification of the densitometry ratio of TFEB normalized to β-actin. Values are means ± S.E.M. ∗∗∗∗p ≤ 0.0001, n = 4. (D) Left panel, immunoblots GBA1 and Cathepsin D. Right panels, quantification of the densitometry ratio of GBA1 and Cathepsin D normalized to their respective β-actin. Values are means ± S.E.M. ∗p ≤ 0.05, ∗∗p ≤ 0.01, n = 3. (C–D) p-value was calculated by Student's t-test.

The results above demonstrate that the activity of the proteasome is altered in cells lacking USP14. USP14 is a DUB with the ability to regulate the ubiquitination status of proteins to affect their degradation or downstream signaling. To screen for other proteins and pathways that could be specifically affected by USP14 deletion, we utilized a proteomic approach using LC-MS/MS and lysates from control and USP14 deficient cells as described in Methods. Proteins with a statistically significant expression changes were assigned to the list of Differentially Expressed Proteins (DEPs) and were further examined by using the Ingenuity Pathways Analysis (IPA) to illustrate the changes in cellular pathways (Fig. 3A and B). Overall, 356 different canonical pathways were identified by IPA as statistically enriched or downregulated in USP14-deleted cells versus control cells as shown by their respective p-values (Table S1). The analyses revealed that the lack of USP14 is associated with changes in the abundance of several DEPs and cellular pathways, such as that for 26S proteasome degradation, ubiquitin metabolism, and chaperones, HSP90 family (association score = 32) (Fig. 3A). In addition, the top-ranked canonical pathways included the Coordinated Lysosomal Expression and Regulation (CLEAR) signaling (predicted to be activated; positive z-score = 3.162) (Fig. 3B). The CLEAR network is involved in the regulation of lysosomes [51], and IPA analysis predicted activation of the transcription factor EB (TFEB) in USP14-lacking cells, along with an elevation in 11 lysosomal enzymes (Fig. S2). Immunoblotting confirmed higher TFEB levels in USP14 deficient cells compared with controls (Fig. 3C). TFEB is a master regulator of lysosomal biogenesis and functions [[51], [52], [53]] and we observed increased levels of the lysosomal enzymes, Glucocerebrosidase-1 (GBA1) and Cathepsin D in USP14 lacking cells as compared to controls (Fig. 3D). These results reveal a novel crosstalk between reduced proteasome and enhanced lysosomal activities via TFEB regulation that may have functional significance for proteostasis.

### 3.4 Mitochondria are elongated in USP14 deficient cells with an increased oxidative stress

**Fig. 4:**
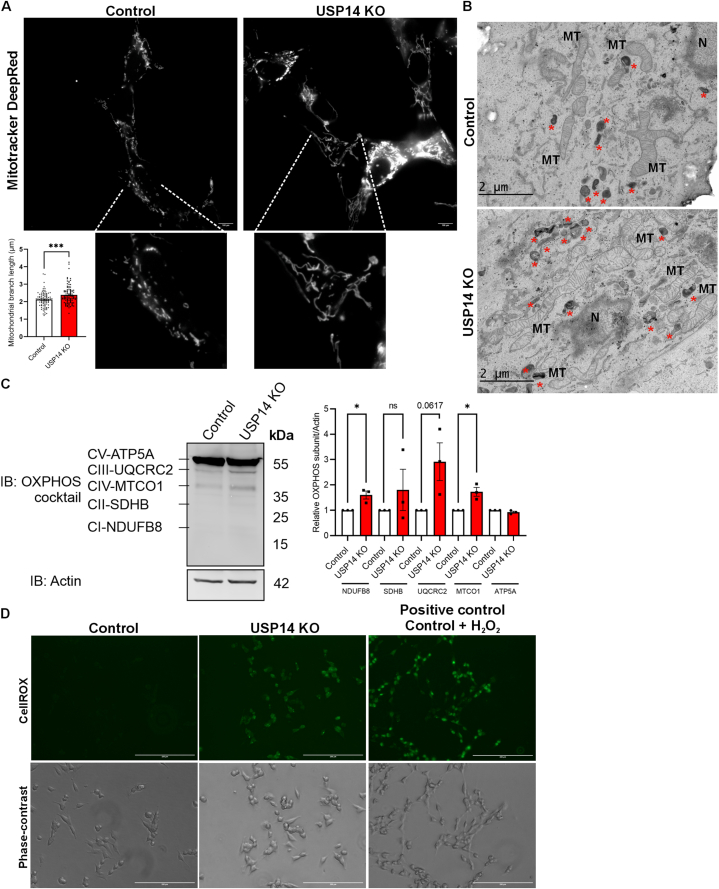
Oxidative stress with elongation of mitochondria in USP14 deficient cells. Control and USP14-deleted cells were cultured and analyzed as indicated below. (A) Live-cell imaging using MitoTracker DeepRed FM and a 100× objective of Nikon-Eclipse Ti-E inverted wide-field microscope equipped with an environmental chamber. MitoTracker DeepRed FM channel (644/665 nm) represented in grayscale. Bottom panels, higher magnification. Left-bottom panel, quantification of mitochondrial branch length in μM from 90 to 100 control and USP14 deleted cells. Scale bar: 100 μM. Typical experiment is shown and was repeated three times with similar results. (B) EM imaging was done as described in Methods. Note elongated mitochondria (MT) in USP14 deleted cells. Red stars ∗ mark the presence of electron-dense lysosomes/autophagosomes/vesicles that were increased in the USP14-deleted cells compared with controls. N represents nuclear compartment. The experiment was repeated with similar results. (C) Left panel, immunoblot using an OXPHOS antibody cocktail. Right panel, quantification of the densitometry ratio of CI-CV subunits (CI-NDUFB8, CII-SDHB, CIII-UQCRC2, CIV-MTCO1 and CV-ATP5A) normalized to β-actin. Values are means ± S.E.M. ∗p ≤ 0.05, ns = not significant. n = 3. p-value was calculated by Student's t-test. (D) Live-cell imaging using CellROX Green and a 20× objective of EVOS FL microscope. Control cells treated with 5 μM H_2_O_2_ for 90 min served as a positive control for oxidative stress. Top, CellROX Green. Bottom, phase-contrast images. Scale bar: 200 μM. Typical experiment is shown and was repeated three times with similar results.

We next assessed possible changes in mitochondria and oxidative stress in SH-SY5Y dopaminergic cells lacking USP14. Live-cell imaging of MitoTracker DeepRed FM staining with quantifications revealed an increase in mitochondrial branch length in USP14-deleted cells compared with controls (Fig. 4A). In accordance with this, transmission electron microscopy (TEM) showed increases in elongated mitochondrial profiles in USP14-deficient cells (Fig. 4B). Furthermore, there was an increase in abundance of dark electron-dense vesicular structures (red asterisk) in USP14-deficient cells that likely represent lysosomes/autophagosomes accumulating in these cells (Fig. 4B).

We next investigated whether there is a change in the expression of oxidative phosphorylation (OXPHOS) subunits in the mitochondria of USP14-deficient cells. Immunoblotting using an OXPHOS antibody cocktail revealed increases in subunits of complex 1 (CI, NDUFB8), complex 3 (CIII, UQCRC2), and complex 4 (CIV, MTCO1) with no changes in subunits of complex 2 (CII, SDHB) or complex 5 (CV, ATP5A) (Fig. 4C, higher exposed IB provided in Fig. S3B). Analysis of the basal mitochondrial oxygen consumption rate using the Seahorse™ assay (Agilent Technology USA) revealed no clear differences between control and USP14-deficient cells (Fig. S3A).

Mitochondria are important sites to produce intracellular reactive oxygen species (ROS) as a byproduct of oxidative phosphorylation reactions mainly at mitochondrial complex 3 [54,55]. Given the changes observed in mitochondrial respiratory complex subunits 1 and 3, we examined the degree of oxidative stress in the USP14-deleted cells using CellROX staining. Live-cell imaging revealed that the amount of ROS was increased in cells lacking USP14 compared to control cells (Fig. 4D). As a positive control, we added H_2_O_2_ which elevated ROS in the control cells (Fig. 4D).

### 3.5 α-Syn and pS129 α-syn are increased in USP14 deficient cells: role of the proteasome and oxidative stress

**Fig. 5:**
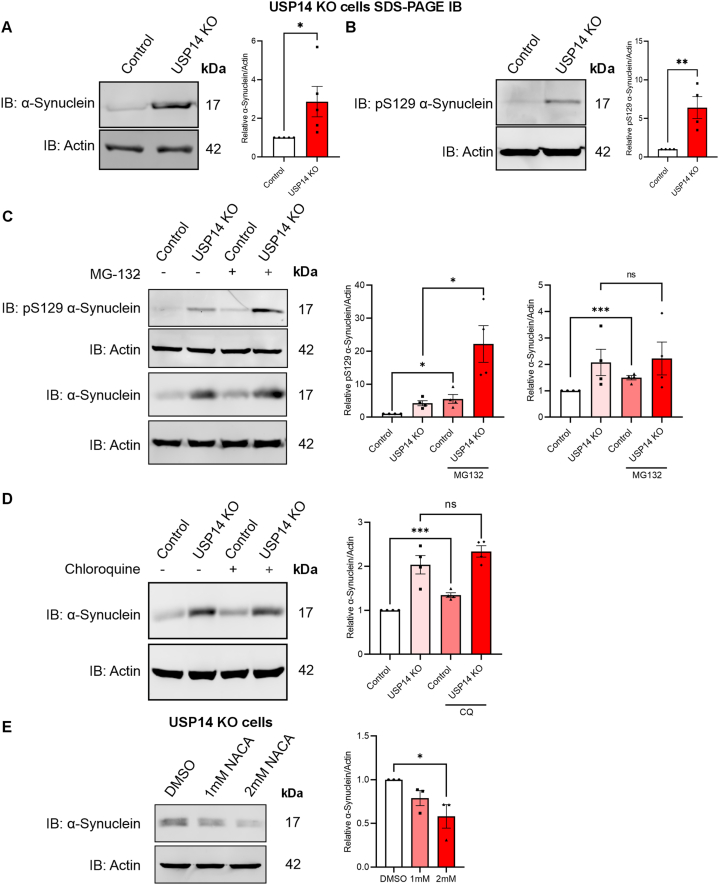
α-Syn and pS129-α-syn levels are increased in USP14-deleted cells. Control and USP14-deleted cells were analyzed as described below. (A–B) Left panels, immunoblots. Note increases in α-syn (A) and pS129 α-syn (B) levels in USP14-deleted cells. Right panels, quantification of the densitometry ratio of α-syn and pS129 α-syn normalized to their respective β-actin. Values are means ± S.E.M. ∗p ≤ 0.05, ∗∗p ≤ 0.01, n = 4–5. (C) Treatment of cells with 20 μM MG132 for 4 h. Left panels, immunoblots of pS129 α-syn and α-syn. Right panels, quantification of the densitometry ratio of pS129 α-syn and α-syn normalized to their respective β-actin. Values are means ± S.E.M. ∗p ≤ 0.05, ∗∗∗p ≤ 0.001, n = 4. (D) Treatment of cells with 200 μM Chloroquine (CQ) for 4 h. Left panel, immunoblots of α-syn. Right panel, quantification of the densitometry ratio of α-syn normalized to β-actin. Values are means ± S.E.M. ∗∗∗p ≤ 0.001, n = 4. (E) Treatment of USP14-deleted cells with 1 or 2 mM N-acetylcysteine-amide (NACA) for 20 h. Left panel, immunoblots. Right panels, quantification of the densitometry ratio of α-syn normalized to β-actin. Values are means ± S.E.M. ∗p ≤ 0.05, n = 3. (A–B) p-value was calculated by Student's t-test and (C–E) one-way ANOVA.

In PD, there is an accumulation of α-syn in vulnerable neurons by mechanisms that are not fully understood. Utilizing immunoblotting, we observed that α-syn levels are increased in SH-SY5Y cells devoid of USP14 compared with control cells (Fig. 5A). Likewise, the level of pS129 α-syn that is linked to PD pathology was also increased substantially in these cells (Fig. 5B). To investigate which pathways are involved in the degradation of α-syn, we used either the proteasome inhibitor, MG132, or the autophagy inhibitor, CQ. The addition of MG132 upregulated the levels of α-syn in control cells (Fig. 5C). However, the accumulation of pS129-α-syn in response to proteasome inhibition was significantly higher and consistent in the USP14-deleted cells compared to that in control cells (Fig. 5C). Inhibiting autophagy with CQ resulted in an increase of α-syn only in the control cells, while USP14-deleted cells did not show any obvious increase (Fig. 5D). These results demonstrate that α-syn and pS129 α-syn accumulate in SH-SY5Y cells lacking USP14, correlative of defective proteasome degradation capacity at basal levels. Further inhibiting the proteasomes with MG132 showed that the lack of USP14 leads to significantly higher increases in pS129 α-syn levels. Albeit the enhanced autophagy in USP14-deleted cells, the α-syn levels did not change significantly upon inhibiting the flux, indicating that autophagy pathways are less important for α-syn clearance in these cells.

To study whether oxidative stress affects α-syn levels in the USP14-deficient cells, we utilized N-acetylcysteine amide (NACA) which is a cell-permeable antioxidant. The addition of 1–2 mM NACA reduced the levels of α-syn in these USP14-ablated cells (Fig. 5E). NACA also exhibited some tendency to reduce pS129 α-syn (data not shown). At the same time, treatment with NACA reduced the levels of ROS in the USP14-ablated cells as shown by CellROX green staining (Fig. S3C). Taken together, these results show that reduced proteasome activity and increases in oxidative stress contribute to elevated α-syn levels in dopaminergic SH-SY5Y cells, and alleviation of oxidative stress reduced these levels.

### 3.6 USP14 is phosphorylated at S143 affecting its structure and functions

**Fig. 6:**
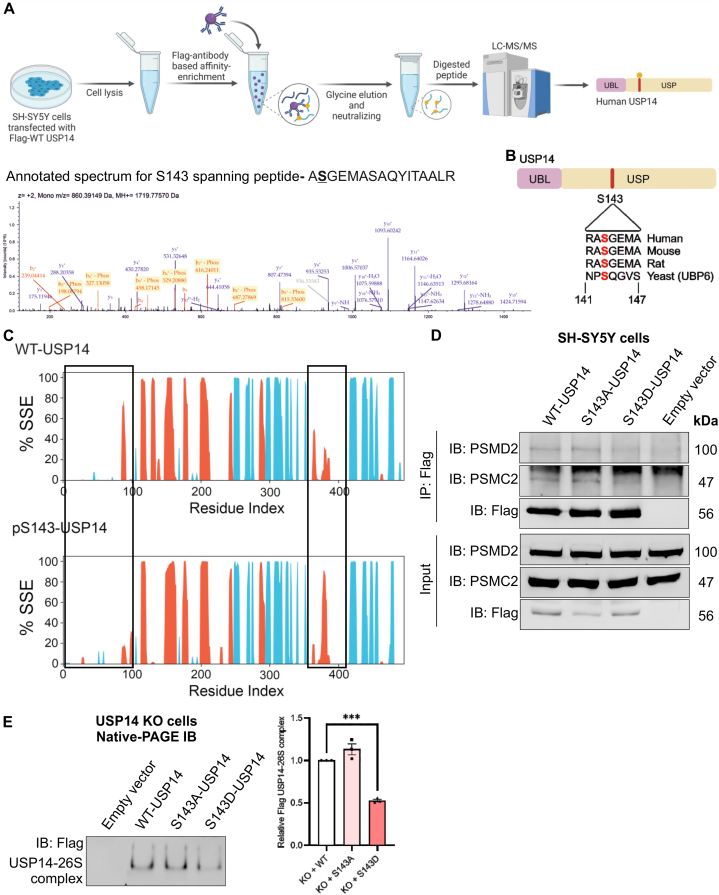
USP14 is phosphorylated at S143 as identified by quantitative mass spectrometry. (A) Workflow for phospho-proteomic experiments. SH-SY5Y cells overexpressing Flag-WT-USP14 were lysed and processed as described in Methods. Immunoprecipitated Flag-WT-USP14 was eluted using glycine, neutralized, enzymatically digested, and processed for LC-MS/MS analysis of phospho-peptides. Below panel, LC-MS/MS annotated mass spectrum of ASGEMASAQYITAALR peptide spanning pS143 residue identified from a representative experiment. The experiment was repeated four times with similar results. (B) Graphical illustration of human USP14 showing the N-terminal ubiquitin-like (UBL) and C-terminal ubiquitin-specific protease (USP) domains. Lower half depicts the sequence alignment and the conservation of the S143 residue across eukaryotes. (C) Protein secondary structure elements (SSE) distribution by residue index throughout the protein structure monitored throughout the MD simulation are shown for WT-USP14 and USP14 phosphorylated at S143 (pS143-USP14). α-helices and β-strands are shown in orange and blue, respectively. The black rectangular boxes indicate the areas with the largest conformational changes. MD simulations were done as described in Methods. (D) SH-SY5Y cells were transfected with Flag-tagged WT-USP14, S143A-USP14 or S143D-USP14 for 24hr, and lysates immunoprecipitated with Flag antibody. The eluates were analyzed by immunoblotting using anti-PSMD2, anti-PSMC2, and anti-Flag antibodies. Input lanes, total cell lysate. A representative experiment is shown and was repeated three times with similar results. Note: Reduced interaction of Flag S143D-USP14 with the 19S RP components PSMD2 and PSMC2. (E) USP14-deleted were reconstituted with Flag-tagged WT-USP14 or S143A-USP14 or S143D-USP14 for 24hr and lysates were analyzed by native-gel electrophoresis followed by immunoblotting for Flag antibody to detect Flag-USP14 bound 26S proteasomes. Right panel shows quantification of the densitometry ratio of Flag-S143A-USP14 or Flag-S143D-USP14 containing 26S complexes normalized to Flag-WT-USP14 containing 26S complexes. Values are means ± S.E.M. ∗∗∗∗p ≤ 0.0001, n = 3. p-value was calculated by one-way ANOVA. Note, the reduction in Flag-tagged S143D-USP14 bound to 26S proteasomes.

As shown above, α-syn levels and oxidative stress are increased in USP14 KO cells indicating a role of USP14 in the viability of SH-SY5Y dopaminergic cells. This raises the question of how USP14 may itself be regulated in these cells. Post-translational modification (PTM) remains as a possible way to influence protein functions and interactions. To examine the presence of PTMs in USP14, we performed a quantitative phospho-proteomic study using overexpression of Flag-WT-USP14 in SH-SY5Y cells, followed by affinity enrichment, and further analysis using LC-MS/MS (Fig. 6A). Phospho-peptide analysis in the Proteome Discoverer 2.5 (Thermo Scientific™) identified putative phosphorylation sites in USP14 at several residues of which S143 phosphorylation was a reliably recurring phosphorylation site. Sequence alignment showed a conservation of this S143 residue in USP14 sequence across eukaryotes including the yeast homolog, UBP6 (Fig. 6B).

To give insights into the structure of USP14, we performed molecular dynamics (MD) simulations of USP14 phosphorylated at the S143 (pS143-USP14) and USP14 mutated at the S143 residue using the Maestro 13.0 InterfaceDesmond package of the Schrödinger LLC. MD simulations with the 30ns length were carried out for each structure. The comparison of changes in the protein secondary structure of WT-USP14 and pS143-USP14 (Fig. 6C) as well as S143A-USP14 and S143D-USP14 mutants (Fig. S4A), suggested that the phosphorylation or point mutation at S143, affects the conformational changes in the UBL domain of USP14. An additional 50ns runs confirmed the stability of the MD simulations and identified results (data not shown).

We next generated phospho-deficient (S to A) S143A-USP14 and phospho-mimetic (S to D) S143D-USP14 mutants to perform biochemical and functional validations. Immunoprecipitation experiments using wildtype USP14 and S143 mutants revealed a reduced binding of the S143D-USP14 mutant to the proteasome 19S RP subunits, PSMD2 and PSMC2 (Fig. 6D). This is in line with the prediction made by MD simulations. Furthermore, USP14-deleted cells were reconstituted with WT-USP14 or S143A-USP14 or S143D-USP14 to analyze the Flag-tagged USP14 bound to 26S proteasomes. Native-PAGE immunoblotting to detect Flag-USP14 containing 26S proteasomes showed reduced Flag-S143D-USP14 containing 26S proteasome complexes (Fig. 6E), which complements our findings that S143D-USP14 binds less to the 19S RP subunits. To investigate whether the phosphorylation at S143 influences the catalytic activity of USP14, we performed a DUB assay utilizing UbVME as a substrate, and parental SH-SY5Y cells expressing either Flag-tagged WT-USP14, S143A-USP14 or S143D-USP14. Immunoblotting for UbVME-bound USP14 revealed that S143D-USP14 had a slightly higher activity compared with WT-USP14 and S143A-USP14 in this assay (Fig. S4B). However, it should be remembered that UbVME is a synthetic substrate and the behavior of USP14 mutants towards natural substrate may be different.

### 3.7 USP14 re-expression reduces ROS, and α-synuclein in USP14-deleted cells

**Fig. 7:**
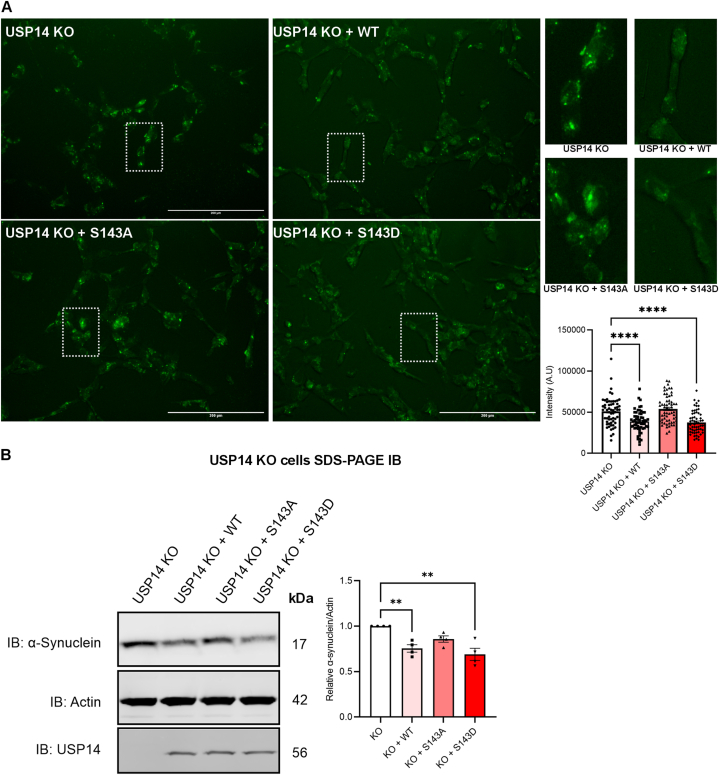
USP14 and S143D-USP14 expression reduces oxidative stress and α-syn levels in USP14-deleted cells. (A–B) Reconstitution experiments. USP14-deleted cells were transfected with plasmids expressing USP14, Flag-tagged wildtype (WT)-USP14 or the mutant USP14, S143A-USP14, and S143D-USP14. 24 h later, cells were analyzed as below. (A) Live-cell imaging of CellROX Green stain. Scale bar: 200 μM. Right panels show representative cells (marked by dotted white rectangle). Note: a reduction in ROS using WT-USP14 and S143D-USP14 mutants but not with the S143A-USP14 mutant. Right panel histogram. Quantification was done by analyzing the intensity of 50–60 cells in each well and plotted as a histogram. A typical experiment is shown and repeated with similar results. Values are means ± S.E.M. ∗∗∗∗p ≤ 0.0001. (B) Top, immunoblots. Right, quantification of the densitometry ratio of α-syn normalized to β-actin. Note: α-syn level is reduced by reconstitution of WT-USP14 and S143D-USP14 in USP14-deleted cells. Values are means ± S.E.M. ∗∗p ≤ 0.01, n = 4. (A–B) p-value was calculated by one-way ANOVA.

To elucidate the functional role of S143 phosphorylation further, we reconstituted WT-USP14, S143A-USP14 or S143D-USP14 in the USP14-deleted cells. Live-cell microscopy using CellROX showed that the WT-USP14 and the S143D-USP14 mutant were able to reduce ROS levels in the USP14-deleted cells (Fig. 7A). Most significantly, the reconstitution of the S143A-USP14 mutant had no significant effect on ROS in the cells (Fig. 7A). To further assess the effect of WT-USP14 and S143 mutants on α-syn, we reconstituted the USP14-deleted cells with WT-USP14, S143A-USP14 or S143D-USP14 and immunoblotted for α-syn (Fig. 7B). Re-expression of USP14 and S143 phospho-mimetic mutant (S143D) reduced the levels of α-syn while the S143A-USP14 mutant exerted no statistically significant effect. In addition, we also observed a small reduction in pS129 α-syn upon reconstitution with WT and S143-USP14 (data not shown). These results in unison indicate that USP14 and S143-USP14 phosphomimetic could directly regulate the levels of oxidative stress, and α-syn in SH-SY5Y dopaminergic neuronal cells.

## 4 Discussion

In the present work, we provide evidence that USP14 is a critical factor for the regulation of proteostasis including α-synuclein clearance and oxidative stress in SH-SY5Y dopaminergic cells. At the molecular level, we observed that deletion of USP14 inhibited the chymotrypsin-like activity of 26S proteasome holoenzyme, and standalone 20S CP, while activating the TFEB-mediated CLEAR/autophagy signaling pathway. USP14-ablated cells exhibited an increase in oxidative stress, which could negatively impact 26S proteasome activity and aggravate mitochondrial dysfunctions. Clearance of α-syn and pS129 α-syn in the USP14-deleted dopaminergic cells was dependent on proteasome activity and on reducing ROS in the SH-SY5Y cells. Using phospho-proteomic analysis we detected that USP14 is phosphorylated at S143 residue, critical for binding to proteasome, and this could be of importance for fine-tuning the USP14-mediated actions in the cell. In line with this, reconstituting WT-USP14 or phospho-mimetic S143D-USP14 mutant counteracted the ROS, and α-syn levels in USP14-ablated cells. This demonstrates a role for pS143-USP14 in regulating oxidative stress and α-syn dynamics in SH-SY5Y dopaminergic cells.

### 4.1 USP14 regulates the 26S proteasome and 20S CP chymotrypsin-like peptidase activity

Previous studies have shown that USP14 is important in regulating the function of 26S proteasome [21,27,28]. Experiments in yeast revealed that the USP14 homolog, UBP6 can induce the activation of the 19S RP ATPase ring, ATP hydrolysis and thus, 26S proteasome peptidase activity [30]. On the other hand, a previous study using purified 26S Proteasomes from USP14-deleted MEF cells showed higher activity for all three peptidases and ATP-hydrolysis [23]. This indicates that the functional interactions of USP14 with the proteasome activities are complex, and context-dependent based on the availability of ubiquitinated substrates and other yet unidentified factors [28].

In our study we observed that the USP14-deleted SH-SY5Y neuronal cells exhibited a loss in 26S proteasome activity along with a reduced amount of the 26S proteasome complex. The basal activity of the free 20S CP was inhibited and this was less pronounced upon stimulating the CP with SDS, indicating a possible increase in free 20S CP gating. The amount of the individual 20S CP subunits was also increased, which could translate to an increased gating of the free 20S CP complex and a related decrease in the standalone 20S CP basal activity. In addition, we did not observe compensatory changes in DUBs that regulate 26S proteasomes, such as in UCHL5 or in the levels of USP14 interacting proteins in 19S RP, PSMD2 or PSMC2. Together these findings support a critical role of USP14 in regulating the 26S proteasomes and the standalone 20S CP. This is in line with the findings in yeast that UBP6/USP14 is important for 26S proteasome activation. These findings provide important insights into the activation of ubiquitin-dependent degradation (26S Proteasome activity) and ubiquitin-independent degradation (standalone 20S CP) by USP14 in SH-SY5Y neuronal cells. Furthermore, previous studied have shown the importance of shuttle factors such as UBQLN2 in the regulation of the proteasomes and in the degradation of misfolded proteins [56]. It would be worthwhile to study the involvement of such shuttle factors in USP14-mediated functions in different cell types, and clearance of aggregation prone proteins, such as α-synuclein/pS129 α-synuclein.

### 4.2 TFEB-mediated CLEAR signaling is increased in USP14-deficient cells

To investigate the pattern of protein targets influenced by USP14 beyond the 26S proteasome network, we performed global proteome profiling in USP14-deleted SH-SY5Y dopaminergic cells. We noted that the TFEB-mediated signaling was increased in USP14-deleted cells as one of the top candidates. TFEB is a master regulator of autophagy-related proteins and lysosomal biogenesis encompassing the previously identified CLEAR network [42,51,52]. TFEB itself is a substrate for the proteasome and undergoes regulation by phosphorylation that controls its nuclear translocation and transcription factor activity [48]. TFEB is further regulated by ubiquitination by the E3 ligase CHIP/STUB1, but less is known about the role of DUBs in TFEB regulation [57]. We show here that TFEB is elevated in USP14-deleted cells together with the downstream ATG8 proteins, and the lysosomal enzymes, β-glucocerebrosidase and cathepsin-D.

Biochemical validations corroborated these findings showing increases in lipidation of LC3B and GABARAP, and autophagic flux in cells lacking USP14. Along with this, we also observed the accumulation of electron-dense structures corresponding to lysosomes and autophagy vesicles in USP14 lacking dopaminergic cells. We interpret the accumulation of these structures as a sign of increased autophagy in the USP14-deficient cells likely as a compensation to proteasome inhibition. The increases in TFEB and autophagy in conjunction with higher levels of α-syn and pS129 α-syn were unexpected but in line with the clearance of these molecules via the proteasomes. The results are also at slight variance with some earlier experiments showing the beneficial effects of overexpressing TFEB in an *in-vivo* model of α-syn toxicity [58]. Moreover, it has been recently reported that an increase in the abundance of lysosomes in brain dopaminergic neurons after injection of α-syn pre-formed fibrils in a model of PD [59]. This resembles the situation in the SH-SY5Y dopaminergic cells devoid of USP14. However, more data is warranted to resolve the roles of lysosomes and autophagy pathways related to α-syn in dopaminergic neurons and in models of PD.

Together the present findings support the view that proteasome and autophagy components exist in close communication in cells as previously observed [46,60]. As to the mechanisms involved, it was found that USP14 can increase levels of the autophagy regulator UVRAG [47] and reduce K63-linked polyubiquitin chains on Beclin-1 [47]. We have further shown that the expression of the W58A-USP14 mutant, incapable of binding to the proteasome, increases GABARAP-positive structures related to selective autophagy in striatal neuronal cells [35]. In addition, inhibition of USP14 in thyroid cancer cells using the compound IU1, augmented LC3B-lipidation, and the autophagy flux [40]. The present results demonstrate that USP14 is important in coordinating responses between proteasomes and autophagy by regulating TFEB and the CLEAR signaling network in the SH-SY5Y dopaminergic cells.

### 4.3 Changes in mitochondria and oxidative stress are present in USP14-deficient cells

Evident from live-cell imaging using MitoTracker and TEM, mitochondria appeared more elongated in USP14-deleted cells compared to controls. The elongated shape of mitochondria could partly be due to changes in cellular proteins regulating the mitochondrial fission and fusion dynamics or lack of autophagosome recruitment to mitochondria. Preliminary data obtained showed changes in the phosphorylation of fission-inducing protein Drp1, possibly causing a reduction in mitochondria fission in the USP14-deleted cells (data not shown). On the functional level, some of the OXPHOS subunits particularly in the respiratory chain complexes 1, 3, and 4 were altered in SH-SY5Y cells devoid of USP14. However, the cells had a largely intact respiratory capacity, as shown by the Seahorse™ assay measuring OCR. We then noted that the levels of cellular ROS were increased in USP14-lacking cells compared with controls as shown by live-cell imaging of CellROX dye. Excessive ROS arising from mitochondrial respiration can lead to oxidative stress that is part of human neurodegenerative disorders including PD and Huntington's disease [61,62]. Oxidative stress can further impact the assembly/disassembly of proteasomes [63] and affect the clearance of oxidative-damaged proteins [64]. As indicated above, USP14 is a regulator of the 26S proteasome, but its potential role in oxidative stress responses is largely unknown. Previous studies showed that proteasomes are involved in the clearance of mitochondrial outer membrane protein as well as misfolded proteins that evade the translocation into the organelle [4,5,65]. This step is also important for priming the rest of mitochondria for clearance via autophagy. Mitochondrial autophagy also contributes to the dynamics and functions of this organelle by clearing the dysfunctional mitochondria [6]. The precise targets for the action of USP14 on mitochondria-related to oxidative stress and its impact on 26S proteasome regulation remains to be studied.

### 4.4 USP14 influences α-syn and pS129 α-syn clearance in SH-SY5Y dopaminergic cells

Recent studies have shown that aggregates of α-syn can disrupt mitochondrial functions and result in oxidative stress in midbrain dopaminergic neurons that are particularly susceptible to degeneration in PD [9,59]. More focus has recently been centered on understanding pS129 α-syn and its role in PD. Studies have identified that an increase in pS129 α-syn correlates with early-onset proteasome defects in dopaminergic neuronal PD models [66]. pS129 α-syn has also been identified to be part of α-syn aggregates and is present in α-syn mutant forms such as A53T [14].

In this work, we observed that α-syn and pS129 α-syn levels are increased in USP14-lacking SH-SY5Y dopaminergic cells mostly due to the observed proteasomal defect. Notably, the increase in pS129 α-syn levels following proteasome inhibition is higher than that of α-syn indicating a difference in the clearing of these molecular species in the SH-SY5Y dopaminergic cells. This in turn could be due to the structure of pS129 α-syn with a higher binding avidity for the proteasome or involve other proteins preferentially interacting with pS129 α-syn. More experiments are currently underway to study this further.

Previous studies have shown that α-syn can undergo ubiquitin-independent degradation via the 20S CP [43]. We observed a defect in 20S CP activity in the USP14-ablated cells that may contribute to the increased α-syn and pS129 α-syn levels.

Oxidative stress is a major component of neurodegenerative diseases including PD. We noted here that the deletion of USP14 in SH-SY5Y dopaminergic cells caused an increase in oxidative stress with elongated mitochondria. These changes occurred concomitantly with increases in the α-syn and pS129 α-syn levels in the cells. This suggests a functional interaction between α-syn and oxidative stress, but the mechanisms involved are likely complex. We noted that the addition of NACA as an antioxidant, decreased ROS, and α-syn levels in the USP14-deleted cells. In addition, reconstituting WT-USP14 and phospho-mimetic S143D-USP14 mutant in USP14-ablated cells lowered α-syn levels. Collectively these findings demonstrate that USP14, and its phosphorylation at S143, together with oxidative stress are involved in the regulation of α-syn clearance in dopaminergic neuronal cells. Recently it was reported that USP14 deficiency can promote the autophagy-mediated clearance of α-syn in mouse models of PD by upregulating S100A8/A9-mediated autophagy processes in microglia cells [31]. In the same study, CSF levels of USP14 were identified to be slightly downregulated in female but not in male PD patients [41]. In the present study we observed that the TFEB/CLEAR signaling and autophagic flux are increased in USP14 deficient SH-SY5Y neuronal cells. Notably, inhibiting autophagy using CQ did not further elevate α-syn in USP14 deficient SH-SY5Y cells. These findings indicate that USP14 may exert differential effects on α-syn regulation by autophagy in neuronal and microglial cells. More work is required to clarify the cell-type specific mechanisms underlying such differences.

### 4.5 Phosphorylation of S143 in USP14 affects its interaction with the proteasome, and the ROS levels in SH-SY5Y cells

PTMs are important determinators in the regulation of protein structure and functions in the cell. Previously it was reported that USP14 can be phosphorylated at serine 432 (S432) in HEK293T cells downstream of the protein kinase Akt [67]. By quantitative phospho-proteomics, we identified that serine 143 (S143) in USP14 was phosphorylated in SH-SY5Y dopaminergic cells, while S432 was not. This data indicates that USP14 can be phosphorylated at different residues, which may vary between cell types and conditions. The catalytic groove pocket in USP14 consists of the catalytic triad of residues: cysteine 114, histidine 435, and aspartic acid 451 [33]. The S143 phosphorylation identified in the present study lies within the region between the α2 and α3 helices of the USP14 catalytic domain (USP-domain) [33]. The MD simulation with WT-USP14, pS143-USP14, and S143-USP14 mutants revealed that the phosphorylation or mutating S143 residue probably affects the conformational changes in the UBL-domain of USP14 [68]. One of most significant differences in the protein secondary structure was observed within the UBL-domain of USP14, that is important for its association with the 26S Proteasomes via PSMD2 [28,32].

To study the functional significance of S143 phosphorylation further, we generated USP14 mutants with either loss of function (S143A) or gain of function (S143D) for functional studies in cells. In line with the MD simulation, we noted that the S143D-USP14 associated less with the 26S proteasome complex, and the 19S subunits PSMD2, PSMC2. Recent NMR study identified that the UBL and USP domains of USP14 could be functionally coupled [69]. In line with this, we identify that phosphorylation of S143 located within the USP domain impacts the UBL-domain and regulate the interaction of USP14 with the 26S Proteasomes.

Furthermore, reconstitution experiments in USP14-deleted cells showed that the S143D-USP14 mutant was effective in reducing ROS, while the S143A-USP14 had no effect. Expression of WT-USP14 also counteracted oxidative stress in USP14-lacking cells, possibly upon undergoing phosphorylation at S143 or other unidentified mechanisms. Further studies are required to investigate the status of USP14 phosphorylation under different physiological conditions. It is also important to characterize the protein kinases and phosphatases involved in USP14 regulation in these SH-SY5Y dopaminergic cells. Given the importance of USP14 in key cellular functions as revealed in this study, phosphorylation of S143 could have a broader function in cell physiology. Elucidating factors with the ability to modulate USP14 and its roles in proteostasis may provide novel targets to interfere with protein aggregates in SH-SY5Y dopaminergic cells and PD.

## 5 Limitations of the study

In this work, we discovered that USP14, via influencing the proteasomes and cellular oxidative stress, can regulate the levels of α-syn/pS129 α-syn in SH-SY5Y dopaminergic neuronal cells. The possible roles of USP14 in affecting other pathogenic variants of α-synuclein, such as the A53T, A30P species of α-syn will require further studies. We further observed that the phosphorylation of USP14 at S143 can affect the binding of USP14 to the proteasome, and reduce oxidative stress and α-syn levels in the cells. The lack of a specific antibody against pS143-USP14 has limited some of our studies and more work is needed in the future on this aspect of the study.

## CRediT authorship contribution statement

**Vignesh Srinivasan:** Writing – review & editing, Writing – original draft, Visualization, Validation, Methodology, Formal analysis, Data curation, Conceptualization. **Rabah Soliymani:** Writing – review & editing, Validation, Methodology, Investigation, Formal analysis, Data curation. **Larisa Ivanova:** Validation, Methodology, Investigation, Formal analysis, Data curation, Conceptualization. **Ove Eriksson:** Writing – review & editing, Validation, Resources, Investigation, Data curation, Conceptualization. **Nina Peitsaro:** Writing – review & editing, Methodology, Investigation, Formal analysis, Data curation. **Maciej Lalowski:** Writing – review & editing, Validation, Methodology, Formal analysis, Conceptualization. **Mati Karelson:** Writing – review & editing, Resources, Methodology, Funding acquisition, Formal analysis, Data curation. **Dan Lindholm:** Writing – review & editing, Writing – original draft, Validation, Supervision, Resources, Project administration, Funding acquisition, Data curation, Conceptualization.

## Data availability

All the proteomics-related dataset has been submitted into MassIVE database under the identifier.

## Declaration of competing interest

The authors declare that they have no known competing financial interests or personal relationships that could have appeared to influence the work reported in this paper.

## References

[1] Hipp M.S., Kasturi P., Hartl F.U. (2019). The proteostasis network and its decline in ageing. Nat. Rev. Mol. Cell Biol..

[2] Ottens F., Franz A., Hoppe T. (2021). Build-UPS and break-downs: metabolism impacts on proteostasis and aging. Cell Death Differ..

[3] Yerbury J.J., Ooi L., Dillin A. (2016). Walking the tightrope: proteostasis and neurodegenerative disease. J. Neurochem..

[4] Bragoszewski P., Turek M., Chacinska A. (2017). Control of mitochondrial biogenesis and function by the ubiquitin-proteasome system. Open Biol..

[5] Kramer L., Groh C., Herrmann J.M. (2021). The proteasome: friend and foe of mitochondrial biogenesis. FEBS Lett..

[6] Uoselis L., Nguyen T.N., Lazarou M. (2023). Mitochondrial degradation: mitophagy and beyond. Mol. Cell.

[7] Calabresi P., Di Lazzaro G., Marino G. (2023). Advances in understanding the function of alpha-synuclein: implications for Parkinson's disease. Brain.

[8] Ganguly U., Singh S., Pal S. (2021). Alpha-synuclein as a biomarker of Parkinson's disease: good, but not good enough. Front. Aging Neurosci..

[9] Henrich M.T., Oertel W.H., Surmeier D.J. (2023). Mitochondrial dysfunction in Parkinson's disease - a key disease hallmark with therapeutic potential. Mol. Neurodegener..

[10] Lehtonen S., Sonninen T.M., Wojciechowski S. (2019). Dysfunction of cellular proteostasis in Parkinson's disease. Front. Neurosci..

[11] Galka D., Ali T.T., Bast A. (2024). Inhibition of 26S proteasome activity by α-synuclein is mediated by the proteasomal chaperone Rpn14/PAAF1. Aging Cell.

[12] Park H., Kam T.-I., Dawson V.L. (2024). α-Synuclein pathology as a target in neurodegenerative diseases. Nat. Rev. Neurol..

[13] Zhao Y., Lin M., Zhai F. (2024). Exploring the role of ubiquitin-proteasome system in the pathogenesis of Parkinson's disease. Pharmaceuticals.

[14] Arawaka S., Sato H., Sasaki A. (2017). Mechanisms underlying extensive Ser129-phosphorylation in alpha-synuclein aggregates. Acta Neuropathol. Commun..

[15] Fujiwara H., Hasegawa M., Dohmae N. (2002). alpha-Synuclein is phosphorylated in synucleinopathy lesions. Nat. Cell Biol..

[16] Ghanem S.S., Majbour N.K., Vaikath N.N. (2022). α-Synuclein phosphorylation at serine 129 occurs after initial protein deposition and inhibits seeded fibril formation and toxicity. Proc. Natl. Acad. Sci. U. S. A..

[17] Kontaxi C., Edwards R.H. (2023). Synuclein phosphorylation: pathogenic or physiologic?. NPJ Parkinsonson Dis..

[18] Ramalingam N., Jin S.X., Moors T.E. (2023). Dynamic physiological α-synuclein S129 phosphorylation is driven by neuronal activity. NPJ Parkinsons Dis..

[19] Nielsen P., Okarmus J., Meyer M. (2023). Role of deubiquitinases in Parkinson's disease-therapeutic perspectives. Cells.

[20] Han R., Wang Q., Xiong X. (2024). Deficiency of parkin causes neurodegeneration and accumulation of pathological α-synuclein in monkey models. J. Clin. Invest..

[21] Hung K.Y.S., Klumpe S., Eisele M.R. (2022). Allosteric control of Ubp6 and the proteasome via a bidirectional switch. Nat. Commun..

[22] Borodovsky A., Kessler B.M., Casagrande R. (2001). A novel active site-directed probe specific for deubiquitylating enzymes reveals proteasome association of USP14. EMBO J..

[23] Kim H.T., Goldberg A.L. (2017). The deubiquitinating enzyme Usp14 allosterically inhibits multiple proteasomal activities and ubiquitin-independent proteolysis. J. Biol. Chem..

[24] Kuo C.L., Goldberg A.L. (2017). Ubiquitinated proteins promote the association of proteasomes with the deubiquitinating enzyme Usp14 and the ubiquitin ligase Ube3c. Proc. Natl. Acad. Sci. U. S. A.

[25] Lee B.H., Lee M.J., Park S. (2010). Enhancement of proteasome activity by a small-molecule inhibitor of USP14. Nature.

[26] Lee B.H., Lu Y., Prado M.A. (2016). USP14 deubiquitinates proteasome-bound substrates that are ubiquitinated at multiple sites. Nature.

[27] Collins G.A., Goldberg A.L. (2017). The logic of the 26S proteasome. Cell.

[28] Goldberg A.L., Kim H.T., Lee D. (2021). Mechanisms that activate 26S proteasomes and enhance protein degradation. Biomolecules.

[29] Zhang S., Zou S., Yin D. (2022). USP14-regulated allostery of the human proteasome by time-resolved cryo-EM. Nature.

[30] Peth A., Besche H.C., Goldberg A.L. (2009). Ubiquitinated proteins activate the proteasome by binding to Usp14/Ubp6, which causes 20S gate opening. Mol. Cell..

[31] Hanna J., Hathaway N.A., Tone Y. (2006). Deubiquitinating enzyme Ubp6 functions noncatalytically to delay proteasomal degradation. Cell.

[32] Collins G.A., Goldberg A.L. (2020). Proteins containing ubiquitin-like (Ubl) domains not only bind to 26S proteasomes but also induce their activation. Proc. Natl. Acad. Sci. U. S. A.

[33] Hu M., Li P., Song L. (2005). Structure and mechanisms of the proteasome-associated deubiquitinating enzyme USP14. EMBO J..

[34] Hyrskyluoto A., Bruelle C., Lundh S.H. (2014). Ubiquitin-specific protease-14 reduces cellular aggregates and protects against mutant huntingtin-induced cell degeneration: involvement of the proteasome and ER stress-activated kinase IRE1alpha. Hum. Mol. Genet..

[35] Srinivasan V., Bruelle C., Scifo E. (2020). Dynamic interaction of USP14 with the chaperone HSC70 mediates crosstalk between the proteasome, ER signaling, and autophagy. iScience.

[36] Liu B., Chen J., Zhang S. (2019). Emerging role of ubiquitin-specific protease 14 in oncogenesis and development of tumor: therapeutic implication. Life Sci..

[37] Liu B., Jiang S., Li M. (2018). Proteome-wide analysis of USP14 substrates revealed its role in hepatosteatosis via stabilization of FASN. Nat. Commun..

[38] Min Y., Lee S., Kim M.J. (2017). Ubiquitin-specific protease 14 negatively regulates toll-like receptor 4-mediated signaling and autophagy induction by inhibiting ubiquitination of TAK1-binding protein 2 and Beclin 1. Front. Immunol..

[39] Sharma A., Alswillah T., Kapoor I. (2020). USP14 is a deubiquitinase for Ku70 and critical determinant of non-homologous end joining repair in autophagy and PTEN-deficient cells. Nucleic Acids Res..

[40] Srinivasan V., Asghar M.Y., Zafar S. (2023). Proliferation and migration of ML1 follicular thyroid cancer cells are inhibited by IU1 targeting USP14: role of proteasome and autophagy flux. Front. Cell Dev. Biol..

[41] Ding L., Lu L., Zheng S. (2024). Usp14 deficiency removes α-synuclein by regulating S100A8/A9 in Parkinson's disease. Cell. Mol. Life Sci..

[42] Settembre C., Medina D.L., Platt F., Platt N. (2015). Methods in Cell Biology.

[43] Machiya Y., Hara S., Arawaka S. (2010). Phosphorylated alpha-synuclein at Ser-129 is targeted to the proteasome pathway in a ubiquitin-independent manner. J. Biol. Chem..

[44] Elsasser S., Schmidt M., Finley D. (2005). Ubiquitin and Protein Degradation, Part A.

[45] Grice G.L., Nathan J.A. (2016). The recognition of ubiquitinated proteins by the proteasome. Cell. Mol. Life Sci..

[46] Kocaturk N.M., Gozuacik D. (2018). Crosstalk between mammalian autophagy and the ubiquitin-proteasome system. Front. Cell Dev. Biol..

[47] Kim E., Park S., Lee J.H. (2018). Dual function of USP14 deubiquitinase in cellular proteasomal activity and autophagic flux. Cell Rep..

[48] Li C., Wang X., Li X. (2019). Proteasome inhibition activates autophagy-lysosome pathway associated with TFEB dephosphorylation and nuclear translocation. Front. Cell Dev. Biol..

[49] Liu W.J., Ye L., Huang W.F. (2016). p62 links the autophagy pathway and the ubiqutin-proteasome system upon ubiquitinated protein degradation. Cell. Mol. Biol. Lett..

[50] Klionsky D.J., Abdelmohsen K., Abe A. (2016). Guidelines for the use and interpretation of assays for monitoring autophagy. Autophagy.

[51] Palmieri M., Impey S., Kang H. (2011). Characterization of the CLEAR network reveals an integrated control of cellular clearance pathways. Hum. Mol. Genet..

[52] Napolitano G., Ballabio A. (2016). TFEB at a glance. J. Cell Sci..

[53] Sardiello M. (2016). Transcription factor EB: from master coordinator of lysosomal pathways to candidate therapeutic target in degenerative storage diseases. Ann. N. Y. Acad. Sci..

[54] Tahara E.B., Navarete F.D.T., Kowaltowski A.J. (2009). Tissue-, substrate-, and site-specific characteristics of mitochondrial reactive oxygen species generation. Free Radic. Biol. Med..

[55] Tirichen H., Yaigoub H., Xu W. (2021). Mitochondrial reactive oxygen species and their contribution in chronic kidney disease progression through oxidative stress. Front. Physiol..

[56] Hjerpe R., Bett J.S., Keuss M.J. (2016). UBQLN2 mediates autophagy-independent protein aggregate clearance by the proteasome. Cell.

[57] Sha Y., Rao L., Settembre C. (2017). STUB1 regulates TFEB-induced autophagy-lysosome pathway. EMBO J..

[58] Decressac M., Mattsson B., Weikop P. (2013). TFEB-mediated autophagy rescues midbrain dopamine neurons from α-synuclein toxicity. Proc. Natl. Acad. Sci. U. S. A..

[59] Geibl F.F., Henrich M.T., Xie Z. (2023). α-Synuclein pathology disrupts mitochondrial function in dopaminergic and cholinergic neurons at-risk in Parkinson's disease. bioRxiv.

[60] Pohl C., Dikic I. (2019). Cellular quality control by the ubiquitin-proteasome system and autophagy. Science.

[61] Reijonen S., Kukkonen J.P., Hyrskyluoto A. (2010). Downregulation of NF-κB signaling by mutant huntingtin proteins induces oxidative stress and cell death. Cell. Mol. Life Sci..

[62] Lin M.T., Beal M.F. (2006). Mitochondrial dysfunction and oxidative stress in neurodegenerative diseases. Nature.

[63] Grune T., Catalgol B., Licht A. (2011). HSP70 mediates dissociation and reassociation of the 26S proteasome during adaptation to oxidative stress. Free Radic. Biol. Med..

[64] Reichmann D., Voth W., Jakob U. (2018). Maintaining a healthy proteome during oxidative stress. Mol. Cell.

[65] Sulkshane P., Duek I., Ram J. (2020). Inhibition of proteasome reveals basal mitochondrial ubiquitination. J. Proteonomics.

[66] McKinnon C., De Snoo M.L., Gondard E. (2020). Early-onset impairment of the ubiquitin-proteasome system in dopaminergic neurons caused by alpha-synuclein. Acta Neuropathol. Commun..

[67] Xu D., Shan B., Lee B.H. (2015). Phosphorylation and activation of ubiquitin-specific protease-14 by Akt regulates the ubiquitin-proteasome system. Elife.

[68] Wang F., Ning S., Yu B. (2021). USP14: structure, function, and target inhibition. Front. Pharmacol..

[69] Salomonsson J., Wallner B., Sjöstrand L. (2024). Transient interdomain interactions in free USP14 shape its conformational ensemble. Protein Sci..

[70] Xicoy H., Wieringa B., Martens G.J. (2017). The SH-SY5Y cell line in Parkinson's disease research: a systematic review. Mol. Neurodegener..

[71] Pham D.D., Bruelle C., Thi Do H. (2019). Caspase-2 and p75 neurotrophin receptor (p75NTR) are involved in the regulation of SREBP and lipid genes in hepatocyte cells. Cell Death Dis..

[72] Roy A., Kucukural A., Zhang Y. (2010). I-TASSER: a unified platform for automated protein structure and function prediction. Nat. Protoc..

[73] Warnecke A., Sandalova T., Achour A. (2014). PyTMs: a useful PyMOL plugin for modeling common post-translational modifications. BMC Bioinf..

[74] Sastry G.M., Adzhigirey M., Day T. (2013). Protein and ligand preparation: parameters, protocols, and influence on virtual screening enrichments. J. Comput. Aided Mol. Des..

[75] Bowers K.J., Chow D.E., Xu H. (2006).

[76] (2006). Proceedings of the 2006 ACM/IEEE Conference on Supercomputing.

[77] Ivanova L., Tammiku-Taul J., García-Sosa A.T. (2018). Molecular dynamics simulations of the interactions between glial cell line-derived neurotrophic factor family receptor GFRα1 and small-molecule ligands. ACS Omega.

[78] Roelofs J., Suppahia A., Waite K.A. (2018). Native gel approaches in studying proteasome assembly and chaperones. Methods Mol. Biol..

[79] Yazgili A.S., Meul T., Welk V. (2021). In-gel proteasome assay to determine the activity, amount, and composition of proteasome complexes from mammalian cells or tissues. STAR Protoc..

[80] Scifo E., Szwajda A., Soliymani R. (2015). Proteomic analysis of the palmitoyl protein thioesterase 1 interactome in SH-SY5Y human neuroblastoma cells. J. Proteonomics.

[81] Valente A.J., Maddalena L.A., Robb E.L. (2017). A simple ImageJ macro tool for analyzing mitochondrial network morphology in mammalian cell culture. Acta Histochem..

[82] Mäkelä J., Tselykh T.V., Kukkonen J.P. (2016). Peroxisome proliferator-activated receptor-γ (PPARγ) agonist is neuroprotective and stimulates PGC-1α expression and CREB phosphorylation in human dopaminergic neurons. Neuropharmacology.

[83] Ignatenko O., Malinen S., Rybas S. (2023). Mitochondrial dysfunction compromises ciliary homeostasis in astrocytes. J. Cell Biol..

[84] Wang L., Yan Z., Vihinen H. (2019). FAM92A1 is a BAR domain protein required for mitochondrial ultrastructure and function. J. Cell Biol..

