# Supplementary material for: USP14 is crucial for proteostasis regulation and α-synuclein degradation in human SH-SY5Y dopaminergic cells
Source: Heliyon. 2025 Jan 23;11(3):e42031. doi: 10.1016/j.heliyon.2025.e42031 (PMC11795799; doi:10.1016/j.heliyon.2025.e42031)
Supplement: Multimedia component 1 [file mmc1.pdf]

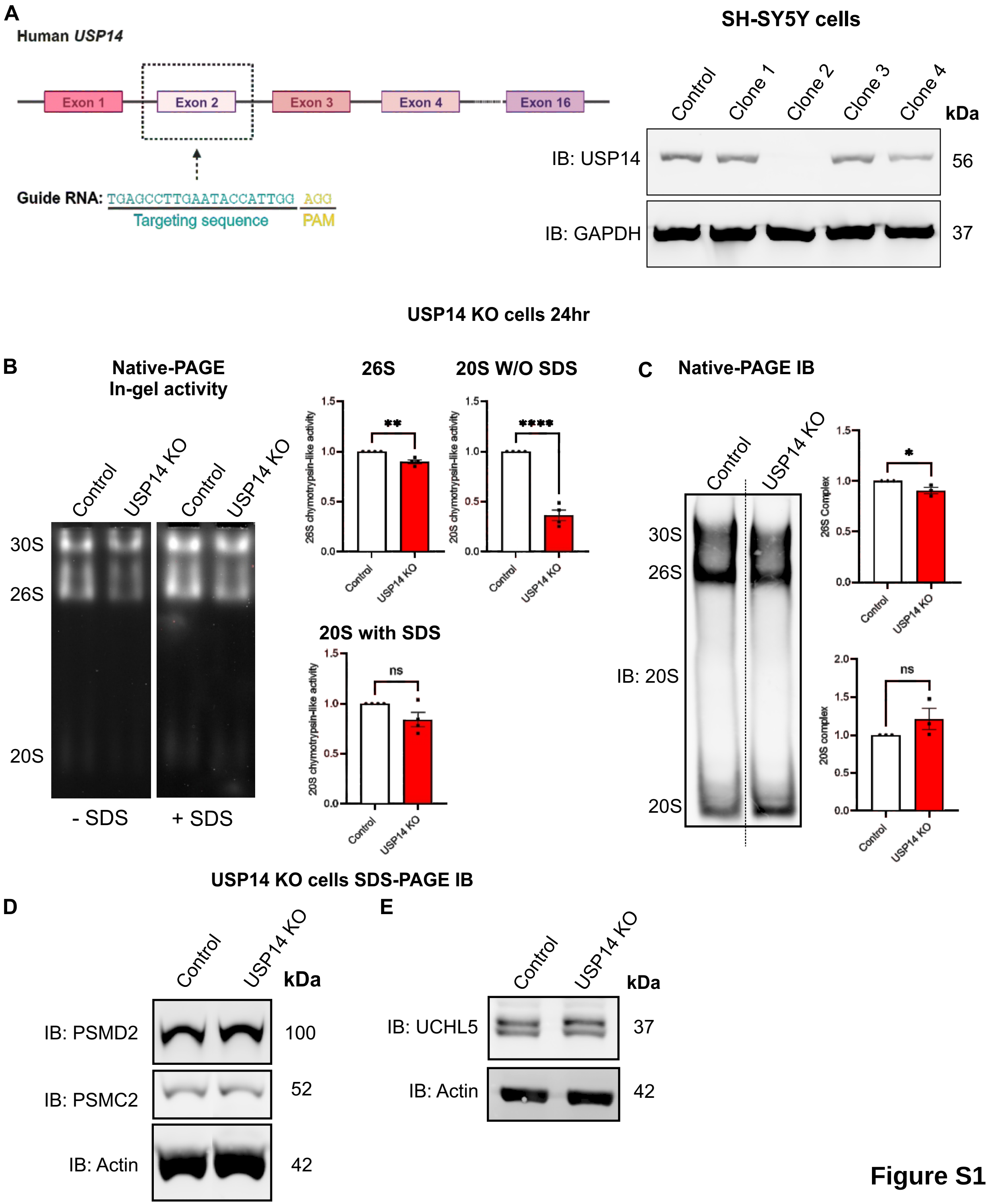
A
Human USP14
Exon 1
Exon 2
Exon 3
Exon 4
Exon 16
Guide RNA: TGAGCCTTGAATACCATTGG AGG
Targeting sequence
PAM
SH-SY5Y cells
Control
Clone 1
Clone 2
Clone 3
Clone 4
kDa
IB: USP14
56
IB: GAPDH
37
USP14 KO cells 24hr
B
Native-PAGE In-gel activity
Control
USP14 KO
30S
26S
20S
- SDS
+ SDS
26S
20S W/O SDS
20S with SDS
26S chymotrypsin-like activity
20S chymotrypsin-like activity
**
****
ns
C
Native-PAGE IB
IB: 20S
26S Complex
20S complex
*
USP14 KO cells SDS-PAGE IB
D
IB: PSMD2
100
IB: PSMC2
52
IB: Actin
42
E
IB: UCHL5
37
IB: Actin
42

Figure S1

CLEAR signaling pathway

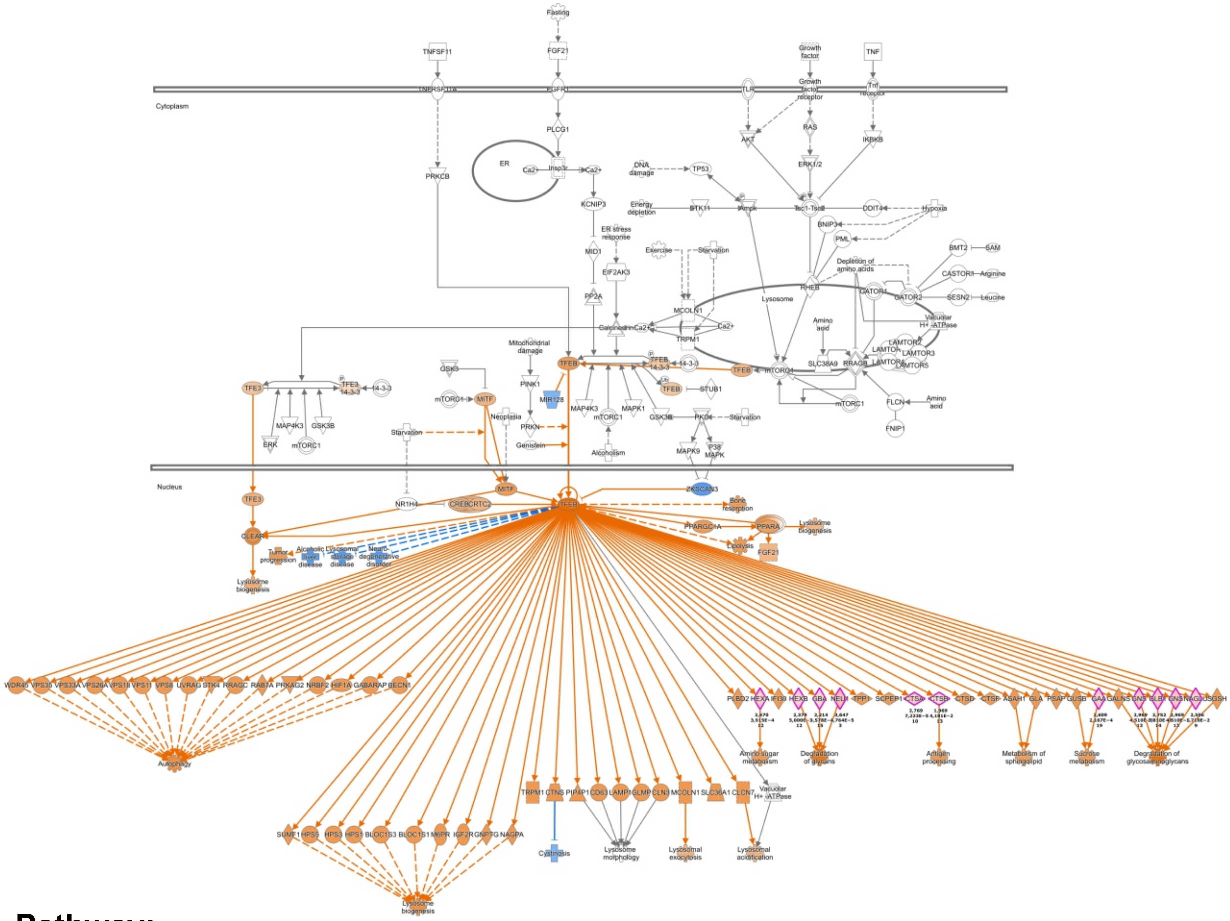

Pathway:

Increased activity

Decreased activity

No trend identified

Inconsistent finding

Direct effect

Indirect effect

Activator

Inhibitor

Identified DEP

Figure S2

Figure S3

A

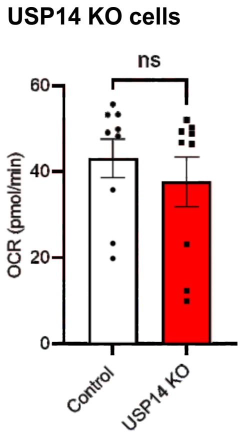

B

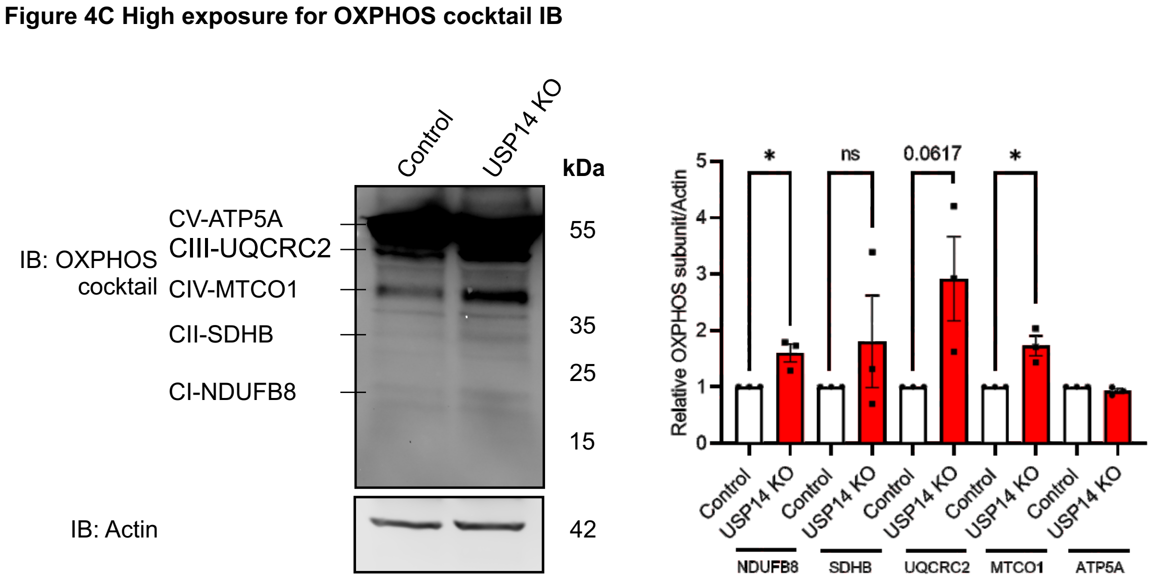

C

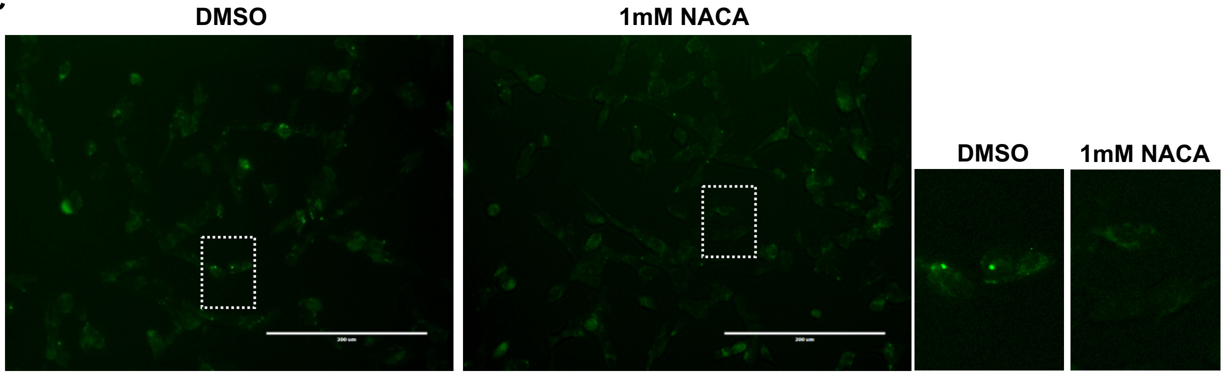

A

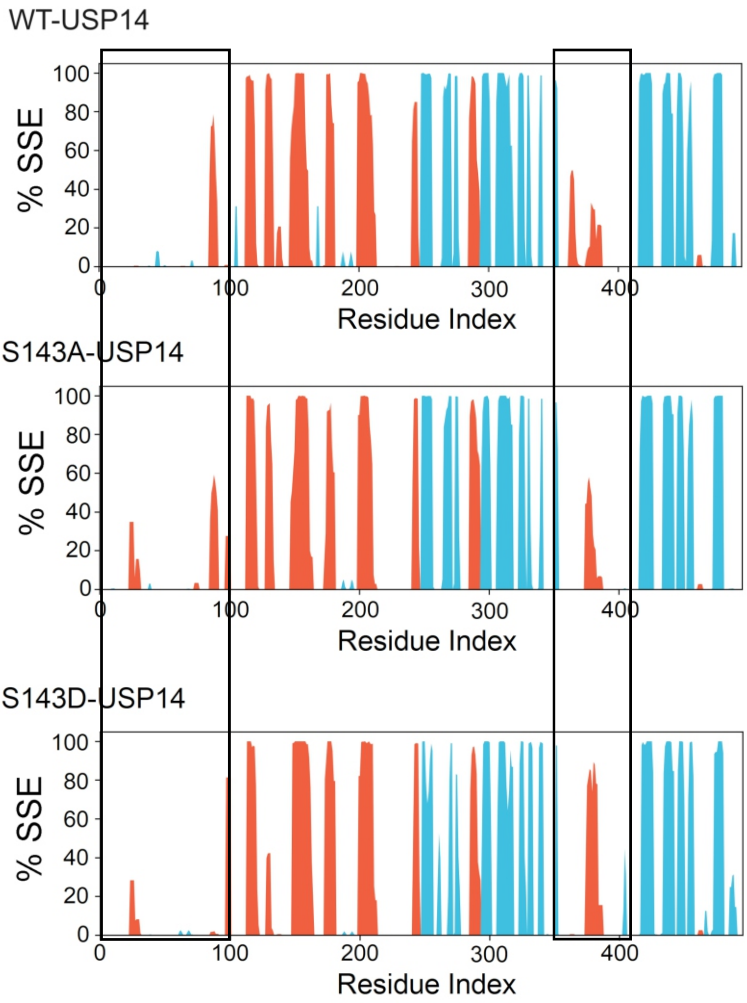
WT-USP14
% SSE
Residue Index
S143A-USP14
% SSE
Residue Index
S143D-USP14
% SSE
Residue Index

B

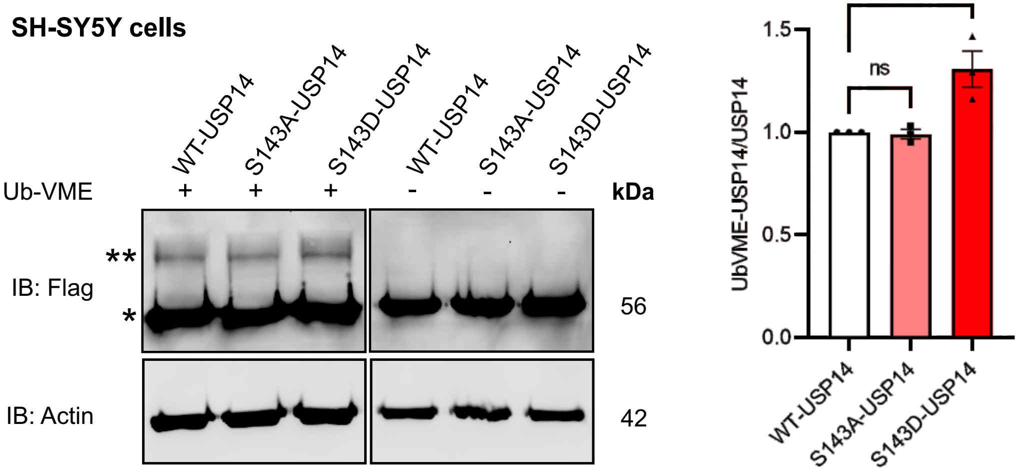
SH-SY5Y cells
WT-USP14
S143A-USP14
S143D-USP14
WT-USP14
S143A-USP14
S143D-USP14
Ub-VME
+ + + - - -
kDa
**
*
IB: Flag
56
IB: Actin
42
*
ns
UbVME-USP14/USP14
WT-USP14
S143A-USP14
S143D-USP14

Figure S4

Supplementary figure legends

Figure S1. 26S Proteasome activity and the complexes are inhibited in USP14 KO cells with no compensatory changes in 19S subunits PSMD2, PSMC2 or UCHL5

(A) Schematic of CRISPR/Cas9 strategy utilized to target exon 2 of *USP14* in human SH-SY5Y cells as detailed in Methods. Right panel shows the single cell clones selected and immunoblotted with anti-USP14 antibody. Cell clones lacking USP14, and control cells were similarly treated and cultivated further for the experiments. GAPDH was utilized as a loading control.

Control and USP14 deleted cells were cultured for 24h (**B and C**) or 48h **(D, E)** and assessed as detailed below

(B) Native-gel electrophoresis followed by in-gel activity assay was done as described in Methods for 26S/30S proteasome and 20S CP linked chymotrypsin-like activity. Left panels, show the UV-exposed native gel with and without addition of SDS to assay the activity of free 20S CP. Right panels, quantification of the densitometry ratio of 26S/30S proteasome, 20S CP activity without (W/O) or with SDS in USP14 deleted cells normalized to controls. Values are means ± S.E.M. $^{**}p \leq 0.01$, $^{***}p \leq 0.001$, $^{****}p \leq 0.0001$, n=4. p-value was calculated by Student's t-test.

(C) Native-gel electrophoresis followed by immunoblotting using a 20S antibody cocktail. Right panels, show quantification of the densitometry ratio of 26S/30S and 20S complex in USP14 deleted cells normalized to controls. Values are means ± S.E.M. ns= not significant. $^{*}p \leq 0.05$. n=3. Dotted line across the image indicate unrelated lanes in-between were removed. p-value was calculated by Student's t-test.

(D-E) Immunoblots for PSMD2, PSMC2 and UCHL5, n=3. Note: No differences in the protein levels of PSMD2, PSMC2 and UCHL5 between controls and USP14-ablated cells.

Figure S2. CLEAR gene network is elevated in USP14-deleted cells

CLEAR gene network is shown with individual proteins identified as DEPs between USP14-deleted and control cells. Legends describing the color-coding is provided on the right.

Figure S3. Mitochondrial OCR is not affected in USP14-ablated cells

(A) Control and USP14 deleted cells were cultured for 48h, and the basal mitochondrial OCR was measured by SeaHorse XF analyzer, values plotted as histogram. No change in mitochondrial basal oxygen consumption rate of USP14 ablated cells.

(B) High exposure image for OXPHOS IB from related to Figure 4C.

(C) USP14-deleted cells were treated with DMSO or 1mM NACA and stained with CellROX green. Live-cell imaging using 20X objective of EVOS FL. Right panels show representative cells (marked by dotted white rectangle). NACA reduced ROS in USP14-deleted cells. Scale bar: 200µM.

Figure S4. S143D influences the UBL domain and S143D-USP14 exhibits a moderate increase in DUB activity

(A) Protein secondary structure elements (SSE) distribution by residue index throughout the protein structure monitored throughout the MD simulation are shown for WT-USP14, S143A-USP14, and S143D-USP14. α-helices and β-strands are shown in orange and blue, respectively. The black rectangular boxes indicate the areas with the largest conformational changes. MD simulations were done as described in Methods.

(B) SH-SY5Y cells were transfected with wildtype (WT)-USP14, S143A-USP14, or S143D-USP14 followed by UbVME substrate assay to assess the catalytic activity of USP14 as described in Methods. Samples were analyzed by immunoblotting using Flag antibody to reveal the amount of UbVME-bound USP14 (marked by **) and unbound USP14 (marked by*). Right panel, densitometry ratio of Flag-USP14 bound to UbVME (**) normalized to Flag-USP14 (*). Values are means ± S.E.M. $^{*}p \leq 0.05$. n=3. p-value was calculated by one-way ANOVA.
